# Molecular insights into land snail neuropeptides through transcriptome and comparative gene analysis

**DOI:** 10.1186/s12864-015-1510-8

**Published:** 2015-04-17

**Authors:** Kevin J Adamson, Tianfang Wang, Min Zhao, Francesca Bell, Anna V Kuballa, Kenneth B Storey, Scott F Cummins

**Affiliations:** Genecology Research Centre, Faculty of Science, Health, Education and Engineering, University of the Sunshine Coast, Maroochydore DC, Queensland 4558 Australia; Institute of Biochemistry & Department of Biology, Carleton University, 1125 Colonel By Drive, Ottawa, ON K1S 5B6 Canada

**Keywords:** Snail, *Theba pisana*, Neuropeptides, Central nervous system, Muscle, Hepatopancreas

## Abstract

**Background:**

Snails belong to the molluscan class Gastropoda, which inhabit land, freshwater and marine environments. Several land snail species, including *Theba pisana*, are crop pests of major concern, causing extensive damage to agriculture and horticulture. A deeper understanding of their molecular biology is necessary in order to develop methods to manipulate land snail populations.

**Results:**

The present study used *in silico* gene data mining of *T. pisana* tissue transcriptomes to predict 24,920 central nervous system (CNS) proteins, 37,661 foot muscle proteins and 40,766 hepatopancreas proteins, which together have 5,236 unique protein functional domains. Neuropeptides, metabolic enzymes and epiphragmin genes dominated expression within the CNS, hepatopancreas and muscle, respectively. Further investigation of the CNS transcriptome demonstrated that it might contain as many as 5,504 genes that encode for proteins destined for extracellular secretion. Neuropeptides form an important class of cell-cell messengers that control or influence various complex metabolic events. A total of 35 full-length neuropeptide genes were abundantly expressed within *T. pisana* CNS, encoding precursors that release molluscan-type bioactive neuropeptide products. These included achatin, allototropin, conopressin, elevenin, FMRFamide, LFRFamide, LRFNVamide, myomodulins, neurokinin Y, PKYMDT, PXFVamide, sCAPamides and several insulin-like peptides. Liquid chromatography-mass spectrometry of neural ganglia confirmed the presence of many of these neuropeptides.

**Conclusions:**

Our results provide the most comprehensive picture of the molecular genes and proteins associated with land snail functioning, including the repertoire of neuropeptides that likely play significant roles in neuroendocrine signalling. This information has the potential to expedite the study of molluscan metabolism and potentially stimulate advances in the biological control of land snail pest species.

**Electronic supplementary material:**

The online version of this article (doi:10.1186/s12864-015-1510-8) contains supplementary material, which is available to authorized users.

## Background

Molluscs are the second largest animal phylum, comprising about 7% of living animals and occupying habitats ranging from high alpine regions to deep sea vents, with a diverse range of lifestyles including predatory, scavenging, herbivorous, detritivorous and filter-feeding [1]. The most abundant class of mollusc are the gastropods, which include the land snails that have evolved independently to life on land. Such a transition has required adaptations towards water saving and breathing dry air.

The central nervous system (CNS), hepatopancreas (or digestive gland) and foot muscle are all key organs controlling or having some influence on a snail’s metabolic rate, whereby a diverse array of cellular modifications is necessary to regulate and co-ordinate changes at the organismal level. Many of these modifications need to be global, and tightly controlled. Such wide ranging control is typically achieved by chemical signalling using neurohormones, many of which are peptides [2]. Neuropeptides, which occur in all animals with a nervous system [3], have been widely studied since the early 1950’s when oxytocin and vasopressin neuropeptides were first identified and characterised [4,5]. Many neuropeptides derive from larger inactive precursors which are proteolytically cleaved into a number of smaller bioactive neuropeptides, generally all the same, but occasionally with different biological actions [3,6].

In molluscs, most neuropeptide research has been on the aquatic gastropods, *Aplysia*, *Lymnaea* and *Lottia.* In the marine gastropod, *Aplysia*, several neuropeptides have been well described that are involved with reproduction and learning [7-9]. In *A. californica,* the best known neuropeptide is FMRF-amide, which appears to provide physiological control of gills [10], and has also been found in heart tissue [11], along with the small cardioactive peptides (SCP_A_ and SCP_B_) which increase heart rate and the amplitude of the beat [11]. The freshwater snail *L. stagnalis* has also been a model for mollusc neuropeptide research where studies have discovered five genes coding for the neuropeptides APGWamide, neuropeptide Y, conopressin, molluscan insulin-related peptide, and pedal peptide that are involved in muscle contraction and modulation in males [12,13]. *In silico* genome and transcriptome database mining have proven effective for high-throughput annotation of the presence and expression of neuropeptides in *Lottia*, as well as in bivalve oysters [9,14]. Less is known about the neuropeptide repertoire of terrestrial pulmonate gastropods, although recently, mass spectrometry has been used to identify and determine changes in neuropeptide profiles in the brain and haemolymph of the snail *Helix pomatia* during activity versus hibernation [15]. In that study, 19 neuropeptides were identified as being more highly produced within the brain of active snails. Meanwhile, during hibernation, 11 neuropeptides were exclusively present [15].

In the current study, we investigated the CNS, hepatopancreas and foot muscle transcriptomes of the land snail *Theba pisana* through gene and peptide analysis. We found numerous neuropeptide precursors that show similarity with other known molluscan neuropeptide precursors, and also demonstrate the existence of numerous other genes that encode peptides destined for secretion. This represents the most extensive analysis of neuropeptide genes and their products in a terrestrial snail.

## Results

### De novo assembly and comparison of *Theba* CNS, foot and hepatopancreas

**Figure 1:**
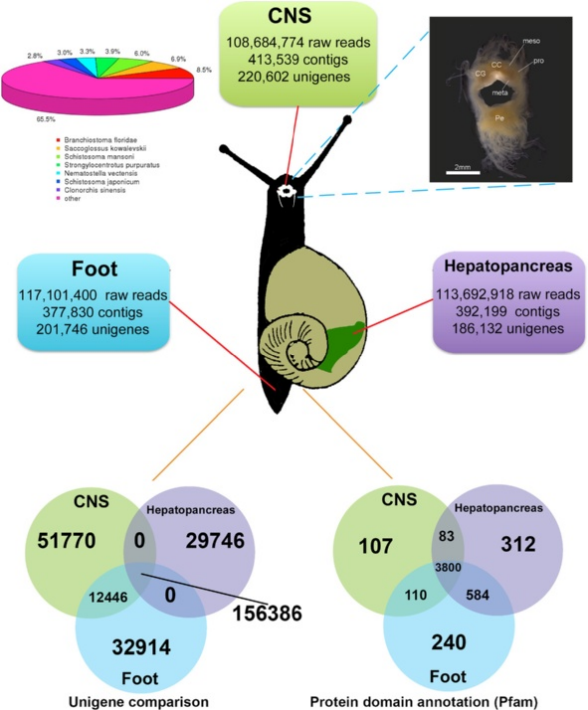
Summary of transcriptome and annotation of genes from *Theba pisana* CNS, hepatopancreas and foot muscle. Figure shows the CNS, including regions of cerebral ganglia (CG), cerebral commissure (CC), mesocerebrum (meso), procerebrum (pro), metacerebrum (meta) and pedal ganglia (Pe). Venn diagrams show comparisons of representative sequences and protein domain annotation between each tissue transcriptome.

Transcript libraries derived from *T. pisana* CNS, foot and hepatopancreas tissues were sequenced using Illumina technologies and assembled. All sequence data was deposited in the NCBI Genbank under SRA file SRP056280. A summary of the number of high quality raw reads, contigs and unigenes for each tissue is shown in Figure 1. The CNS, foot and hepatopancreas transcriptomes encoded 220,602, 201,746, and 186,132 unigenes, respectively. A unigene is typically interpreted as representing a single genomic locus; hence, these groups represent the first comprehensive non-redundant putative transcript database for *T. pisana*. Collectively, contigs and unigenes could be assembled into a total of 250,848 consensus sequences ranging in size from 200 to 26,246 bp; size distribution for the pooled tissue transcriptome is shown in Additional file 1: Figure S1. Approximately 156,386 representative sequences are shared within the three transcriptomes (Figure 1). Although CNS has more unique unigenes compared to foot or hepatopancreas, there was no substantial difference based on protein functional domain annotation (Figure 1). In total, there were 24,920 CNS proteins, 37,661 foot proteins and 40,766 hepatopancreas proteins annotated, with 5236 unique Pfam domains. Combining the unigene and Pfam comparison results, the data revealed that the three tissues have a moderate number of tissue-specific transcripts that encode proteins that play similar functions in cellular processes.

### Functional annotation

*T. pisana* sequences were annotated against protein databases (Nr, Nt, SwissProt, KEGG, GO and COG) using BLASTX (E-value < 0.00001). From the 250,848 consensus sequences, 69,799 (27.8%) had at least one hit. The sequence names and annotation information of all sequences are provided in Additional file 2: Table S1. The majority of transcripts had either a significant match with those from the pacific oyster (*Crassostrea gigas*) or did not match to any known genes; this result is most likely due to insufficient sequences being available in public databases from phylogenetically closely related species. The annotation rate in our study is comparable to those that have been reported in previous *de novo* transcriptome sequencing studies for molluscs [16,17].

**Figure 2:**
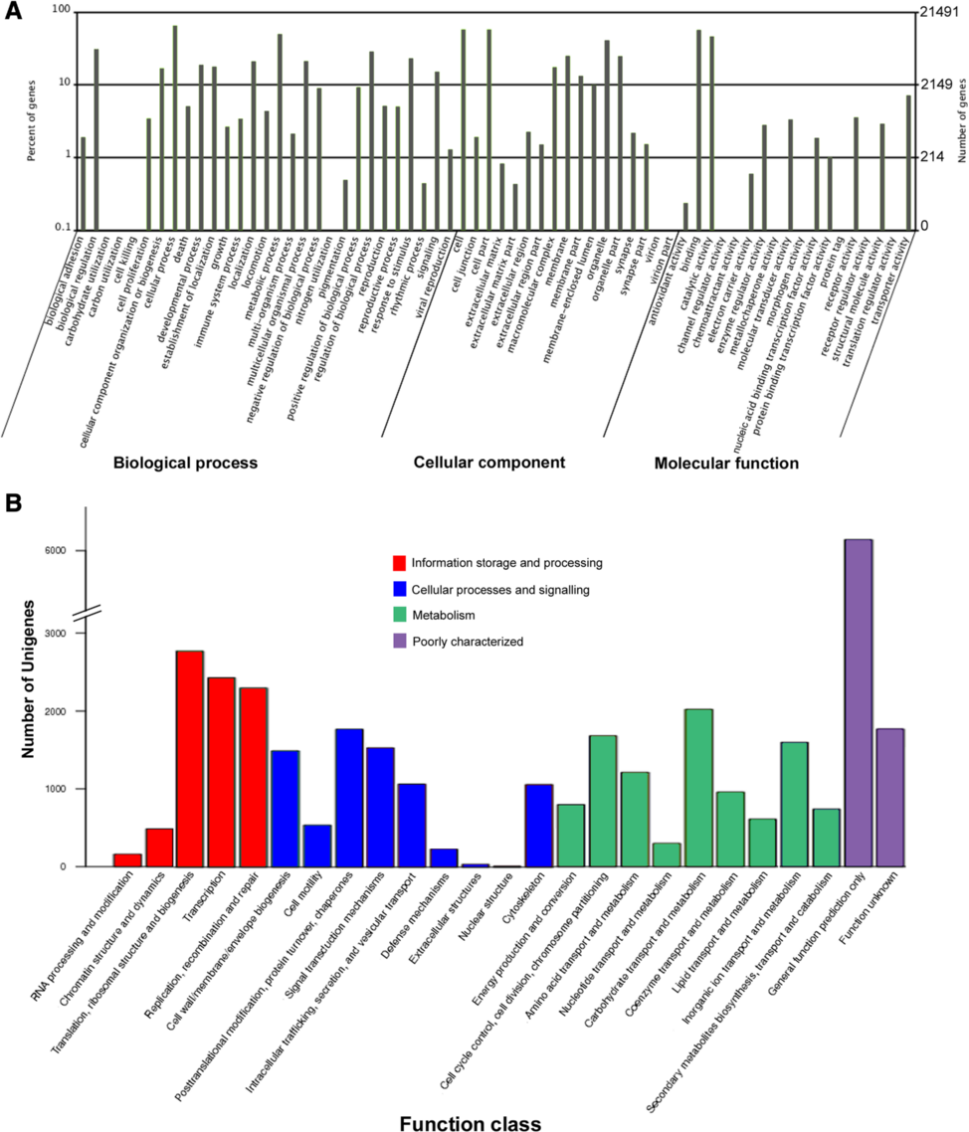
Predicted functional analysis. (**A**) Analysis of gene ontology to classify functions to *T. pisana* genes (**B**) Graph showing the assignment of the *T. pisana* unigenes to categories of the eukaryotic cluster of orthologous groups of proteins (COG). The main COG categories (functional classes) are represented with different colours.

Gene ontology was performed to classify functions to *T. pisana* genes (Figure 2A). Of these, 77,715 transcripts were assigned to functional categories of ‘Biological Process’ (48.5%), 27,187 to ‘Molecular Function’ (34.5%) and 55,346 to ‘Cellular Component’ (17%). Functional annotation of all transcripts combined against the cluster of orthologous groups (COG) database is shown in Figure 2B. These were assigned to four primary COG classes: Information storage and processing (8156 transcripts), Cellular processes and signalling (7756 transcripts), and Metabolism (9932 transcripts) as well as Poorly characterized genes (7,911 transcripts). The COG functional classification demonstrates that the most abundant classification is “general function prediction only”, followed by “translation, ribosomal structure and biogenesis” and “transcription”.

Transcript abundance was determined based on fragments per kilobase of exon per million fragments mapped (FPKM; Additional file 1: Figure S2 and Additional file 2: Table S1). In the CNS, of the 50 most abundant transcripts (besides ribosomal), 22% encoded for neuropeptides such as insulin, neuropeptide Y, myomodulin and achatin, whereas unannotated transcripts comprised 40% of transcripts. In the hepatopancreas, many of the top 50 abundant transcripts (minus ribosomal) encoded for catabolic enzymes including cathepsin peptidase, serine peptidase, chitotriosidase-1, myosinase, lysozyme, while 20% were unannotated including the most abundant transcript (Unigene64357_All). In the foot muscle, the epiphragmin-encoding transcripts were most prominent. Common transcripts that were of high abundance in all three transcriptomes included those encoding a polyubiquitin protein, heat shock protein 70 and an elongation factor 1 alpha.

### Annotation of mollusc proteins secreted from CNS in *Theba pisana*

**Figure 3:**
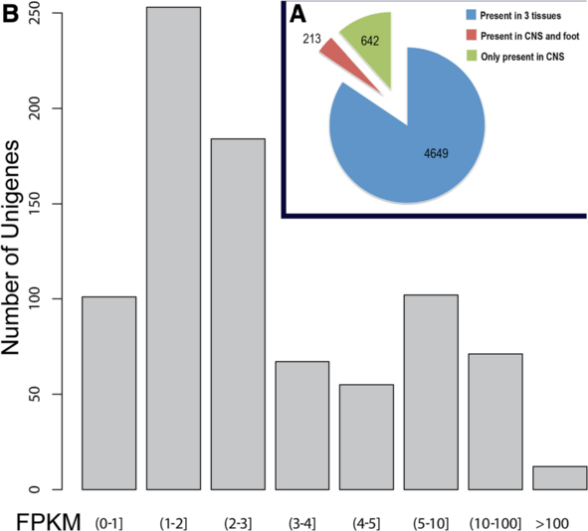
Distribution and level of gene expression, based on FPKM. (**A**) Relative distribution of CNS predicted secreted proteins. (**B**) Relative expression level of secreted protein transcripts in the CNS.

Investigation of the *T. pisana* CNS transcriptome, including identification of those precursors containing signal peptides and no transmembrane domains, revealed that *Theba* might contain as many as 5504 precursor proteins that are destined to secrete peptides (Additional file 3: Table S2). Of those, 4649 putative proteins are expressed in all three transcriptomes (Figure 3A). Another 213 were expressed in both CNS and foot, while there appeared to be no overlap in expression between CNS and hepatopancreas. The remaining 642 proteins were expressed exclusively in CNS. On the basis of a fold change higher than 5, we defined a list of 849 putative secreted proteins in CNS. As shown in Figure 3B, the expression FPKM for the majority of the putative secreted peptides were less than 3. Of those where expression was >100 and 10–100, 81% and 22% are designated as encoding hypothetical proteins, respectively (Additional file 3: Table S2).

**Figure 4:**
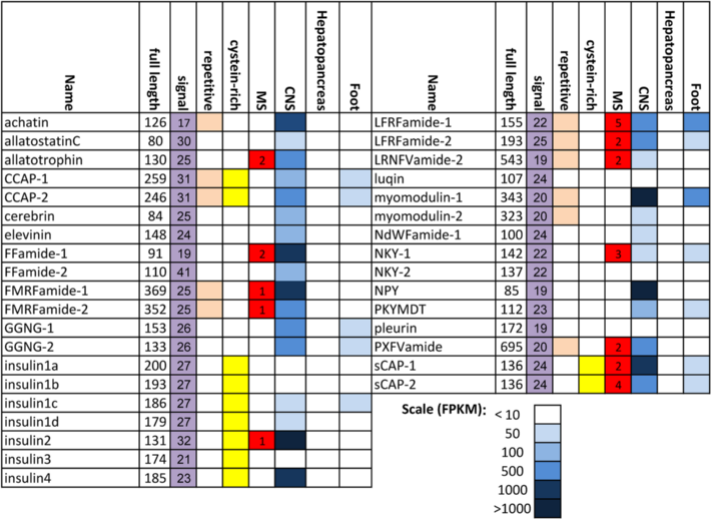
Summary of molluscan neuropeptides, their distribution and characteristics identified in *Theba pisana*.

Of the putative secreted proteins in the CNS, full-length precursors were identified in *T. pisana* for 35 known molluscan neuropeptides (Figure 4 and Additional file 4), including isoforms and insulin that in molluscs can be secreted from neural tissue. In Figure 4, a “white” cell is defined as indicating only a trace amount or no transcript (<10). In general, for these neuropeptide transcripts, abundance was most prominent within neural tissue, particularly for insulin2, NPY, myomodulin1 and achatin (Figure 4 and Additional file 1: Figure S3).

**Figure 5:**
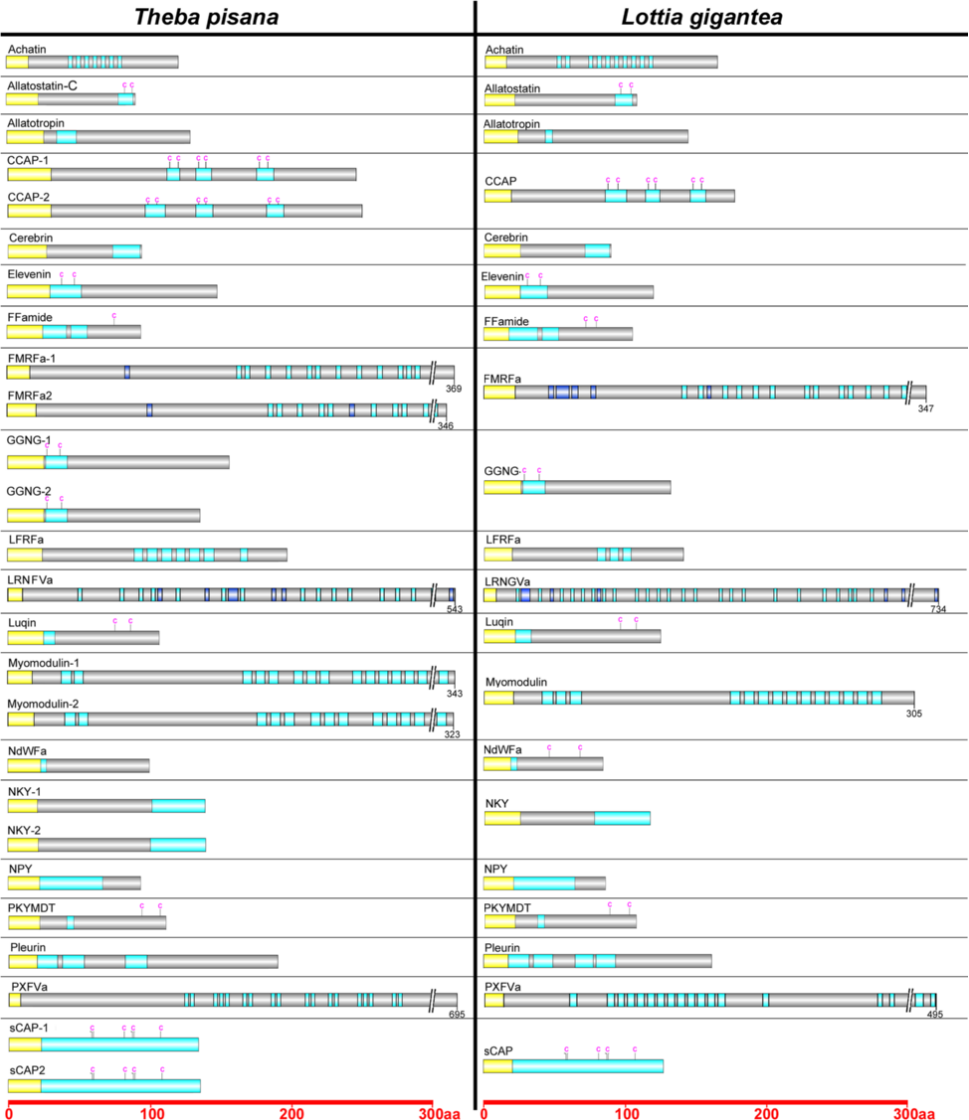
Schematic representation showing organisation of *Theba pisana* and *Lottia gigantea* neuropeptides. Yellow, signal peptide; blue, bioactive peptides; C, cysteine. Scale bars at bottom show length in amino acids (aa).

Full-length precursors for some of these neuropeptides were also found to be of intermediate abundance in foot muscle while absent from the hepatopancreas. Peptide extraction followed by LC-MS analysis was used to confirm the usefulness of transcriptome-derived protein libraries and complement *in silico* neuropeptide predictions (Figure 4 and Additional file 1: Figure S4). Figure 5 shows the organisation of *T. pisana* neuropeptide precursors compared with previously identified homolog precursors of a marine gastropod, the limpet *Lottia gigantea*, with general high conservation in the spatial organisation of bioactive peptide sequences, as well as precursor size, cleavage sites and position of cysteine residues. Several of these neuropeptides were targeted for more in-depth tissue expression analysis.

**Figure 6:**
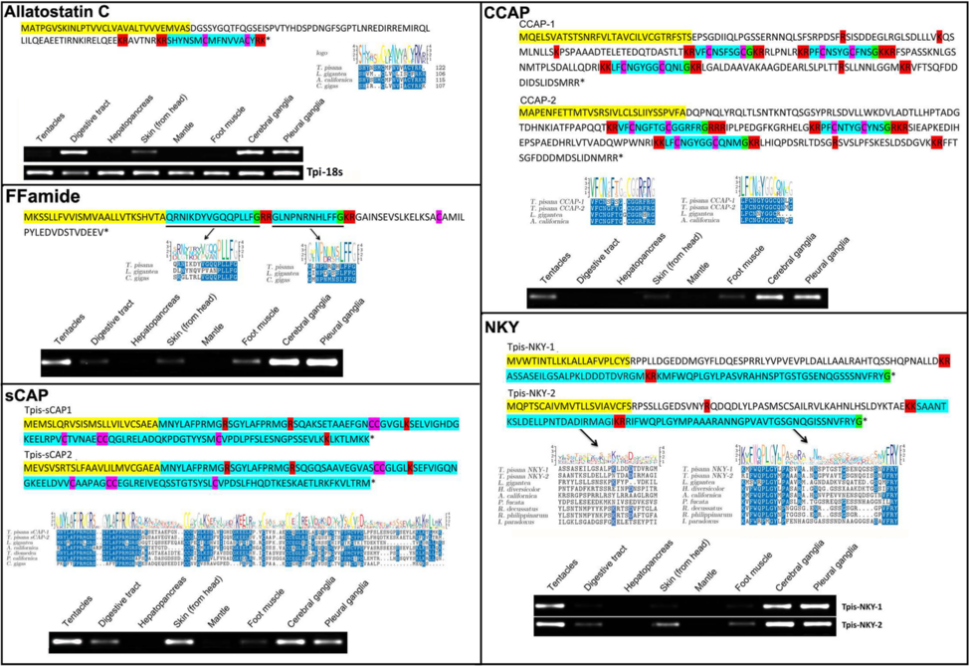
Characterization of *Theba pisana* allatostatin C, CCAP, FFamide, sCAP and NKY. Each protein is represented by; (1) primary amino acid sequence with signal sequence (yellow), cleavage sites (red), cysteine residues (pink) and bioactive region (blue); (2) multiple amino acid sequence alignment with other gastropod and bivalve molluscs; and (3) RT-PCR expression of the neuropeptide gene within various tissues.

The Tpi-Allatostatin C precursor comprised 80 amino acids, including signal peptide and cleavage sites to release the 15 amino acid active allatostatin C located in the C-terminal region of the precursor (Figure 6). Most similarity with Allatostatin C from other species was found within the bioactive peptide, primarily at the N-terminal SH and spatial conservation of two cysteine residues that likely form disulfide bonds to help stabilise the peptide. *Tpi-allatostatin C* was only identified in the CNS transcriptome, at a low expression level (Figure 4) but RT-PCR analysis showed that it was expressed not only in cerebral and pleural ganglia but also in *T. pisana* digestive tract and skin tissue (Figure 6). A representative RT-PCR of the 18 s rRNA gene is also shown, confirming the integrity of all tissue RNA. Two Crustacean cardioactive peptide (CCAP) precursor transcripts were identified in *T. pisana*, both encoding 3 CCAP moieties and showing very high amino acid identity with *Lottia* and *Aplysia* homologs (Figure 6). *Tpi-CCAP1* and *2* transcripts were identified in CNS and foot muscle transcriptomes, with CNS CCAP-2 being more abundant (Figure 4). RT-PCR demonstrated that *Tpi-CCAP1* is additionally expressed within sensory tentacles. The *Tpi-FFamide* transcript encoded 2 different amidated peptides; an LLFamide peptide and an LFFamide peptide, which show conservation with known homologs from other species within the C-terminal regions (Figure 6). Mass spectral analysis of the CNS of active *T. pisana* identified peptides corresponding to both LLFamide and LFFamide. Both *Tpi-FFa* transcripts were identified in the CNS transcriptome, with FF-1 very highly expressed (Figure 4). *Tpi-FFa-1* was additionally expressed within the tentacles, digestive tract, skin and foot muscle tissues, despite not being identified from the foot muscle transcriptome.

Two *Tpi-NKYamide* transcripts were identified that encode neuropeptide Y (NKY) precursors of 142 and 137 residues, each containing cleavage sites to release two bioactive peptides, a 24-residue peptide and the 38-residue NKY peptide (Figure 6). Little amino acid conservation exists for the 24-residue peptide with other species. For the 38-residue NKY precursor, conservation with other species exists primarily within the N- and C-terminal regions, with very limited amino acid identity in the middle region. Mass spectral analysis of the CNS identified peptides corresponding to both NKY precursor peptides. Both *Tpi-NKY1* transcripts were observed in the CNS and foot muscle transcriptomes, while *Tpi-NKY2* was detected in very low amounts in the CNS transcriptome only. RT-PCR found that *Tpi-NKY1* wa*s* also present in the tentacles and *Tpi-NKY2* was additionally found in the tentacles, digestive tract, skin and foot muscle tissue. Two *Tpi-sCAP* transcripts were identified that both encode precursor proteins of 136 amino acids, including spatial conservation of putative cleavage sites and cysteine residues (Figure 6). High conservation between species was observed in the N-terminal region of the bioactive peptide. Mass spectral analysis of the CNS identified peptides corresponding to both sCAP precursor peptides. Both *Tpi-sCAP* transcripts were identified in high abundance within the CNS, and less in the foot muscle, while RT-PCR showed expression of *Tpi-sCAP1* in the tentacles, digestive tract and skin.

**Figure 7:**
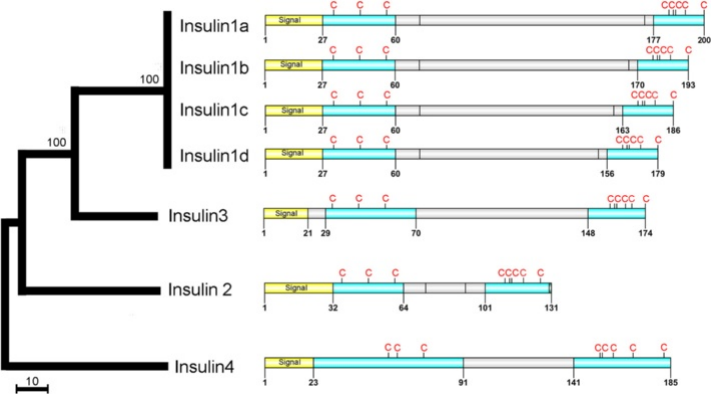
Identification and characterization of *Theba pisana* insulins. Phylogeny of 7 insulins identified and representative schematic showing the organisation of the signal peptides (yellow), bioactive peptides (blue), cleavage sites at dibasic and tetrabasic amino acids (vertical lines) and cysteine (C) residues. Scale bar represents amino acid substitutions.

We identified from the three *T. pisana* transcriptomes, 7 transcripts with strong homology to insulins. Multiple sequence comparison and phylogeny demonstrated 4 groups of insulin-like peptides with strong bootstrap support; *Tpi-insulin1*_*a-d*_*, Tpi-insulin2, Tpi-insulin3 and Tpi-insulin4* (Figure 4, Figure 7 and Additional file 1: Figure S5). *Tpi-insulin* precursors vary in size from the largest at 200 amino acids (*Tpi-insulin1*_*a*_) to the smallest at 131 amino acids (*Tpi-insulin2*). Consistent with other species, the organisation of *T. pisana* insulins consists of a “B peptide” with 3 cysteines, followed by a “C peptide” and then a C-terminal “A peptide” containing 5 cysteines. Only the Tpi-insulin2 A peptide contains a glycine residue site for amidation. *Tpi-insulin2* and *Tpi-insulin4* were highly expressed in the CNS, while only *Tpi-insulin1*_*c*_ could be identified in another transcriptome (foot muscle). No further RT-PCR analysis was performed to investigate insulin expression in other snail tissues.

### Prohormone convertases

**Figure 8:**
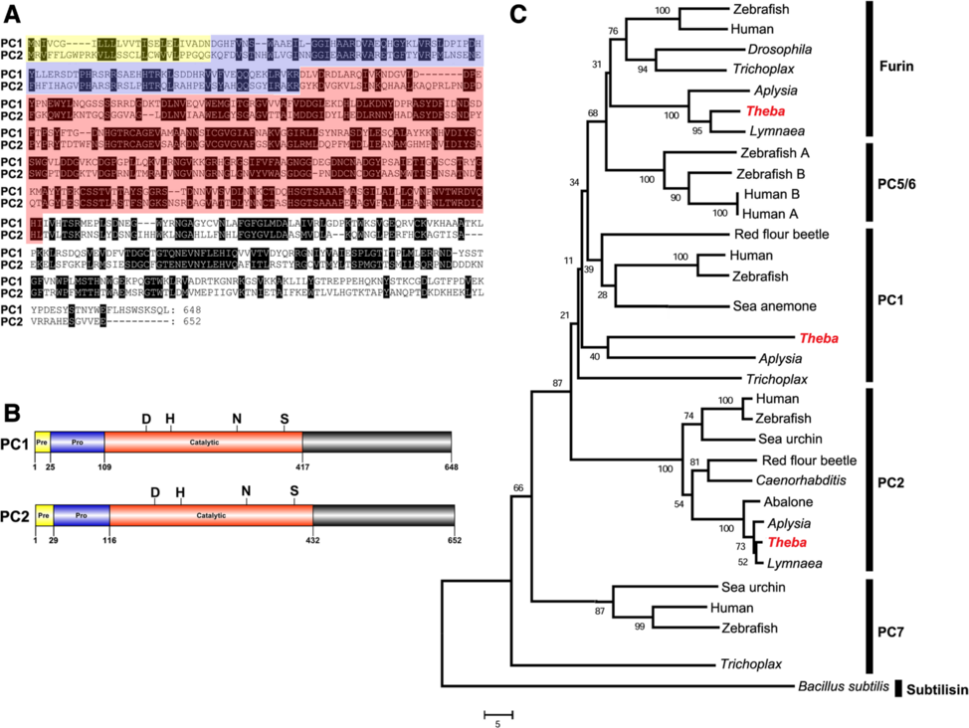
Identification and characterization of *Theba pisana* prohormone convertases (PCs). (**A**) Amino acid alignment between PC1 and PC2 shows strong homology, especially within the catalytic region, shown schematically in (**B**); shown are signal (pre; yellow), pro domain (blue) and catalytic region (red). (**C**) *T. pisana* PCs cluster within the PC1, PC2 and furin-type convertases. Scale bar represents amino acid substitutions. Genbank accession numbers for all PCs are listed in Additional file 4.

We identified transcripts in each transcriptome database encoding enzymes that cleave precursor proteins, including the prohormone convertase 1 (PC1) and PC2 (Figure 8A, B and Additional file 1: Figure S6). *Tpi-PC1* and *Tpi-PC2* encode precursors of 648 and 652 amino acids, respectively, and contain regions typical of these processing enzymes, including signal, pro and catalytic regions that are required for cleavage of dibasic amino acids. Another type of convertase, the *Tpi-furin* was also identified that encodes a partial-length furin (554 amino acids) with a large 3’ untranslated region. Multiple sequence alignment and phylogeny support their enzyme grouping (Figure 8C).

## Discussion

In this study, we extend on our understanding of gastropod biology by investigating transcriptomes of the land snail, *Theba pisana*. Deep transcript sequencing followed by *in silico* predictions and mass spectral analysis enabled the identification of numerous molluscan genes in this snail, including previously unknown neuropeptides. This greatly enhances our ability to utilise this species as an experimental model in neuroscience and metabolism, with the potential to enable development of molecular tools that could manipulate the physiology and behaviour of this agricultural and horticultural pest species.

Deep sequencing was performed to enable the assembly of three transcriptomes representing the CNS, hepatopancreas and foot muscle. Since gene expression levels may vary considerably depending on animal behavioural and physiological state, tissue samples for RNA transcriptome sequencing were combined from stages representing active and aestivated, reproductive and juvenile snails. With over 300 million raw reads providing approximately 200,000 unigenes for each tissue, this ensured a comprehensive analysis of tissue genes. *De novo* assembly was necessary since no annotated reference is currently available for *Theba* or any other helicid snail. BLAST analysis showed that the majority of genes matched most closely with oyster, *Crassostrea gigas*, which represents the most expansive list of molluscan genes within the NCBI database due to the release of its genome and associated transcriptomes [18]. As a bivalve, *C. gigas* belongs to a different taxonomic group, thus our submission of the *Theba* transcripts derived from this study ensures that helicid snail gene sequences are now far better represented in the NCBI databases. Abundant unannotated transcripts suggest that there may be numerous helicid-specific sequences, or this species is at least considerably different to those currently annotated.

Gastropods have long been used in experimental neuroscience projects, providing a rich source for neurological discoveries. The vast majority of these studies have used marine and freshwater gastropods, including *Aplysia* and *Lymnaea*. In *Lymnaea stagnalis*, two separate studies analysed neural transcriptomes, first using normalized EST screening to identify 7,712 distinct transcripts [19], which was then expanded via Illumina sequencing to provide 116,355 contigs [20]. A large-scale neural EST screen of *Aplysia* CNS has revealed 175,000 transcripts, including 19,814 unique (at the time) neuronal gene products [21]. In our study, Illumina sequencing enabled a more comprehensive identification of *T. pisana* CNS transcripts, providing 220,602 unigenes. Several of the *Theba* neuropeptides identified have been shown to be significantly up- or down-regulated in the brain or hemolymph of hibernating *Helix pomatia* when compared to active individuals [15]. For instance, levels of peptides derived from the FMRFa, myomodulin, and probably LRNFVa precursors are much more abundant when *H. pomatia* are in the hibernation state, suggesting they may be critical for initiating and/or maintaining metabolic arrest. The CNS, hepatopancreas and foot muscle are crucial tissues regulating how snails initiate and maintain deep hypometabolic states, and therefore our comprehensive transcriptomes and associated transcriptome-derived protein databases should provide a useful platform to use for comprehensive gene-associated hypometabolic studies in these animals [22-24].

It has been speculated that in invertebrates, insulins, which are found within neurons, may have a role in neurotransmission [25]. Similar to vertebrate homologs, the invertebrate insulin precursor contains A and B chains, linked by a connecting (C) peptide. This C peptide is cleaved from the precursor and the A and B chains connect by cysteine bridges to form the mature insulin peptide [26]. In many cases, following cleavage of the precursor, the mature peptides are subjected to post-translational modification such as glycosylation, C-terminal amidation, acetylation, phosphorylation or sulfation [3]. Insulin precursors are known to undergo a series of processing events that yield the functional insulin, consisting of A and B peptides bound by intermolecular disulfide bonds. We found that *T. pisana* have 7 insulins, with each sharing similar attributes common to all other insulins. The largest reported molluscan insulin has been the *Aplysia* insulin; its involvement in regulating glucose metabolism was determined through the demonstration that *Aplysia* insulin can reduce hemolymph glucose levels [27]. Of all the *T. pisana* insulins, the *Tpi-insulin2* transcript appears to be the most highly expressed insulin in the CNS. Future studies are planned to determine the molecular role for this insulin.

This study shows that a prohormone convertase is present within a land snail. The ability for a cell to secrete peptides relies on specific molecular secretory machinery, including processing enzymes that contribute to the synthesis of a mature bioactive peptide. In molluscs, PCs have been reported for *Lymnaea*, *Aplysia* and *Haliotis* [28-30]. The *Lymnaea* PC2 gene is exclusively expressed in the neuroendocrine system and may be present as two alternatively-spliced isoforms, similar to the *Haliotis* genes which are differentially expressed in neural tissues [28]. We found only one PC2 gene (as well as one PC1) in *T. pisana*, indicating that one of each is sufficient in this species to perform precursor cleavages, including cleavage of allatostatin C, FFamide and sCAP.

For gastropods, the hepatopancreas and foot muscle are major sites for lipid metabolism and storage, and therefore are critical during periods of starvation and hypometabolism [23,31]. The hepatopancreas is also a major source of digestive enzymes and is involved in nutrient absorption, food storage and excretion [32]. For that reason, it is not surprising that metabolic enzyme genes dominate transcripts annotated within the hepatopancreas. The foot muscle, on the other hand, contains numerous epiphragmin gene transcripts that encode for the epiphragmin protein, a major constituent of the epiphragm mucus that seals off the aperture during hibernation and aestivation [33]. The dried epiphragm is necessary to enable moisture retention while still allowing for gaseous exchange [34]. In the snail *Cernuella virgata*, a major source of epiphragmin appears to be the mucous glands [33], while our study also implicates the foot muscle as a rich source of this protein. The foot contains its own type of mucus-secreting cells that were likely incorporated into our foot tissue transcriptome. During prolonged hypometabolism, muscle disuse atrophy (possibly related to reactive oxygen species) may be a major issue and we expect that future use of this transcriptome may enable us to establish molecular pathways by which these snails circumvent damage during hibernation and aestivation. Enzymes that have been implicated as key proteins in metabolic depression in other gastropod species, such as pyruvate kinase, phosphofructokinase and glutamate dehydrogenase (GDH) [35,36] are present in *T. pisana* transcriptomes and also represent obvious targets for future metabolic studies in this species. Furthermore, GDH activity can be as much as three times higher in aestivating snails than in active animals; GDH is crucial to amino acid metabolism and in aestivating snails helps to funnel nitrogen into urea biosynthesis which leads to the elevated urea in body fluids that contributes to desiccation resistance [22,37].

The identification of genes involved in the maintenance of land snails, as reported here, has numerous implications. Prior to this study, there was a lack of gene data for land snail species, despite their importance in ecological functioning. *Theba pisana* is one of several snails native to the Mediterranean region that are now established as pests in grain crops, pastures and vineyards in southern Australia, where they not only eat seedling crops and pastures, but also clog harvesting equipment and contaminate grain harvests [38-40]. The current study provides a foundation for further studies into the manipulation of pest snail behavioural/physiological states that could lead to novel treatments to control their populations. For example, as demonstrated in insect pests [41,42], neuropeptide mimetic analogs hold potential for blocking or overstimulating receptors. There are also a number of other invasive snail species that have become major pests worldwide, including aquatic snail pests in reservoirs in Israel [43], the New Zealand mud snail *Potamopyrgus antipodarum* in Europe, Australia, Japan and North America [44], the golden apple snail, *Pomacea canaliculata*, in rice fields throughout Asia [45], and the giant African snail *Achatina fulica* throughout the tropics and subtropics [46]. In addition, the common garden snail *Cornu aspersum* Müller now inhabits large areas of Western Europe in man-made habitats [47] and is an introduced pest of many areas of the world, including North America [48], Australia [49] and New Zealand [50].

## Conclusion

This study has identified the genes expressed in three tissues of *Theba pisana* and identified numerous CNS neuropeptide gene products, confirming their expression *in vivo* by RT-PCR and mass spectrometry. Investigations can now progress into defining the function of gene products.

## Methods

### Animals and tissue collection

*Theba pisana* were collected from agricultural sites surrounding Warooka, located on the Yorke Peninsula, South Australia in early spring (September). Snails were transported to the University of the Sunshine Coast (USC) and housed within purpose-built enclosures. Snails were provided with water and food (cucumber and carrot) *ad libitum* and maintained at room temperature.

Active snails were conditioned by feeding and supplied with water daily over 7 days. Snails to be used for aestivation experiments were placed into glass jars without food or water and kept in an incubator on a cycle of 12 h at 30°C with light, 12 h at 20°C dark, to emulate South Australian summer conditions. The positions of the snails were marked on the jars after 14 days. Any snails that had not moved following a further 21 days were deemed to be in aestivation. To obtain waking snails, aestivated snails were sprayed with water and harvested as soon as they became active (10–60 min). CNS, hepatopancreas (digestive gland) and foot muscle tissue was dissected out of *T. pisana* and immediately frozen. In each case, tissue from active, waking and aestivating snails was combined to maximise transcript representation from each metabolic state.

### RNA isolation and transcriptome sequencing

RNA was extracted from tissue using TRIzol Reagent (Invitrogen), as per the manufacturer’s protocol. Following extraction, RNA was assessed for quality by visualisation on a 1.2% denaturing formaldehyde agarose gel, quantified using a Nanodrop spectrophotometer (Thermo scientific). For complimentary DNA (cDNA) synthesis, RNA samples were subjected to oligo-dT selection for mRNA purification and fragmented into small fragments. Fragmented RNA samples were subsequently repaired before adapter ligation. Suitable fragments were selected and reversed-transcribed into double-stranded cDNAs. The cDNA libraries were constructed by PCR amplification using random hexamer primed cDNAs. Finally, the samples were used for library construction and sequenced using an Illumina HiSeq 2000 sequencing (BGI, Hong Kong).

### Gene ontology and identification

*De novo* assemblies for each tissue type were performed by Trinity software [51] using trimmed reads from Illumina sequencing. The assembler was run with the parameter sets as following: seqType, fq; minimum kmer coverage = 4; minimum contig length of 200 bp. Sequences without Ns and which could not be extended on either end were defined as unigenes. When there were several samples from the same species, TGICL [52] was used to assemble all the unigenes from different samples to form a single set of non-redundant unigenes. After clustering, the unigenes were divided into two classes, clusters with the prefix CL, and singletons with the prefix Unigene.

Transcripts were annotated with the databases of NR, NT, Swiss-Prot, KEGG, COG, and GO, using BLAST and BLAST2GO software. Relative abundances of all transcripts among different tissues were estimated by SOAP software version 2.21. Searches of molluscan neurohormone precursors were also conducted by performing tBLASTn search of all tissue transcripts, which were reported by previous “omics” analysis studies [53-57]. BLAST searches were performed in CLC Main Workbench (Version 6.0). All hits were then analyzed manually with their orthologous peptides from various species and then their structures were characterized.

Analysis of protein identity/similarity between different protein receptors was performed by protein alignment using clustalW2 (http://www.ebi.ac.uk/Tools/msa/clustalw2/). The percent identity was calculated as the number of identical amino acid residues, as indicated by "*" symbol in the Clustal output, divided by the total number of amino acid residues of the longest sequence (×100). All hits (lowest E-value) were run through the SignalP 3.0 website for signal peptide prediction with the Neural Networks algorithm (Center for Biological Sequence Analysis, Technical University of Denmark, Lyngby, Denmark; http://www.cbs.dtu.dk/services/SignalP/ ref). Proteolytic cleavage sites as well as post-translational modifications were predicted based on homology to other known peptides and the Neuropred tool (neuroproteomics.scs.illinois.edu/neuropred.html).

### Protein comparison and Pfam domain annotation

To generate the most complete possible set of *T. pisana* peptides, we predicted protein-coding regions using OrfPredictor [58] by default parameters on each tissue-specific assembly. We only retained the predicted longest ORFs and translated those into amino acid sequences over 30 amino acids. These sequences represented the full transcriptome-derived proteome. These were grouped into identical amino acid sequences using BLASTCLUST (BLAST score-based single-linkage clustering, [ftp://ftp.ncbi.nih.gov/blast/documents/blastclust.html]). We required the minimal length coverage threshold to be 0.7, which means that the minimum alignment length should cover at least 70% of full length of the shortest member in a group of sequences. For each resulting sequence clustering group, the longest amino acid sequence was chosen as the representative. The first optimal protein sequence in the sequence group was selected if multiple peptides had identical amino acid lengths. To provide an overview for the biological function, we annotated all the predicted proteins using the Pfam database (version 27.0). HMMSEARCH [42] was adopted to associate proteins with Pfam domains. We used a threshold of 0.01 for the e-value to identify reliable hits.

### Neuropeptide prediction and sequence analysis

Neuropeptides are generally secreted out of the cell, which is facilitated by signal peptides in the premature protein form. To systematically identify putative neuropeptides in the CNS of *T. pisana*, we initially utilized four bioinformatics tools on all putative CNS proteins to predict the presence of a signal peptide (SignalP 3.0 [59] and PrediSi [60]) and any transmembrane domain (TMHMM 2.0 [61] and HMMTOP 2.1 [62]). For all these tools, we used default settings and parsed the results using in-house perl script. Then, resulting proteins were used as input to the NeuroPred tool to predict cleavage products. Schematic diagrams of protein domain structures were prepared using Domain Graph (DOG, version 2.0) software [63]. Protein sequences from *T. pisana* were aligned against a database prepared from known sequences from NCBI (January, 2014) using the MEGA 5.1 [64] platform with the clustalW protocol utilising the Gonnet protein weight matrix. Neighbour-joining trees were generated based off these alignments. Unrooted trees were generated with 1000 bootstrap trials and presented with a cut-off bootstrapping value of 50.

### Tissue distribution of selected genes of interest

Tissue was dissected out of 4 animals and each tissue type was pooled. Tissue types collected were tentacles, digestive tract, hepatopancreas, skin from head, mantle, foot muscle, cerebral ganglia and pleural ganglia. Total RNA was extracted from the 8 tissue types using TRIzol Reagent (Invitrogen, Catalogue # 15596–018) as per the manufacturer’s protocol. RNA extracts were assessed using agarose gel electrophoresis, and quantified using a Nanodrop 2000 spectrophotometer (Thermo scientific). First-strand cDNA was synthesised using QuantiTect Reverse Transcription kit (Qiagen) as per the manufacturer’s protocol. To normalise cDNA for each sample, equal quantities of extracted RNA were used as templates. Amplification of cDNA was carried out using the *Taq* F1 DNA Polymerase kit (Fisher Biotec) in a total volume of 25 μl. The PCR reagent mix was prepared as recommended by the manufacturer, containing 1× PCR reaction buffer, 2 mM MgCl_2,_ 0.2 mM each dNTPs, 0.2 μM forward primer and reverse primer (sequences available upon request), 1 unit of Taq F1 DNA polymerase, 17.9 μl water and 1 μl cDNA template. A negative control, substituting water for template, was included in all PCR experiments. PCR was performed in a Kyratec Model SC200 thermal cycler. Cycling parameters were: initial denaturation at 95°C for 5 min, followed by 35 cycles of denaturation at 94°C for 20 s, annealing at 55°C for 30 s, and extension at 72°C for 1 min. Final extension was at 72°C for 10 min, then held at 10°C. Primers used were designed from sequences for *Theba pisana* 18 s rRNA gene and 6 peptide precursor genes (available upon request). A positive control reaction using cDNA from mixed tissues was also performed for each PCR reaction.

### Peptide isolation from CNS and nanoHPLC, mass spectrometry LC-ESI-QTOF peptide identification

CNS were dissected out and combined from active and aestivated (3 weeks without movement) mature *T. pisana*, then immediately frozen in liquid nitrogen prior to storage at −80°C until use. Frozen samples of CNS were ground to a powder under liquid nitrogen in a mortar, then quickly weighed and homogenized in extraction buffer (90% methanol, 9% glacial acetic acid in deionized water) in a 1:5 w:v ratio. Crude extracts were then sonicated with three pulses, 30 s each, and centrifuged for 20 min (16,000 × g, 4°C). Supernatant was collected and lyophilised.

The CNS extracts were analyzed by LC-MS/MS on a Shimadzu Prominance Nano HPLC (Japan) coupled to a Triple-ToF 5600 mass spectrometer (ABSCIEX, Canada) equipped with a nano electrospray ion source. Aliquots (6 μl) of each extract were injected onto a 50 mm × 300 μm C18 trap column (Agilent Technologies, Australia) at 30 μl/min. The samples were de-salted on the trap column for 5 minutes using solvent A [0.1% formic acid (aq)] at 30 μL/min. The trap column was then placed in-line with the analytical nano HPLC column, a 150 mm × 75 μm 300SBC18, 3.5 μm (Agilent Technologies) for mass spectrometry analysis. Peptide elution used a linear gradient of 1-40% solvent B [90:10 acetonitrile:0.1% formic acid (aq)] over 35 min at 300 nl/minute flow rate, followed by a steeper gradient from 40% to 80% solvent B over 5 min. Solvent B was then held at 80% for 5 min to wash the column and then returned to 1% solvent B for equilibration prior to the next sample injection. The ionspray voltage was set to 2400 V, declustering potential (DP) 100 V, curtain gas flow 25, nebuliser gas 1 (GS1) 12 and interface heater at 150°C. The mass spectrometer acquired 500 ms full scan TOF-MS data followed by 20 by 50 ms full scan product ion data in an Information Dependent Acquisition (IDA) mode. Full scan TOFMS data was acquired over the mass range 350–1800 and for product ion ms/ms 100–1800. Ions observed in the TOF-MS scan exceeding a threshold of 100 counts and a charge state of +2 to +5 were set to trigger the acquisition of product ion, ms/ms spectra of the resultant 20 most intense ions. The data was acquired and processed using Analyst TF 1.5.1 software (ABSCIEX, Canada).

Proteins were identified by database searching using PEAKS v7.0 (BSI, Canada) against the protein database built from the CNS transcriptome. Search parameters were as follows: no enzyme was used; variable modifications included methionine oxidation, conversion of glutamine to pyroglutamic acid, deamidation of asparagine and amidation. Precursor mass error tolerance was set to 20 ppm and a fragment ion mass error tolerance was set to 0.05 Da. Maximum expectation value for accepting individual peptide ion scores [−10*Log(*p*)] was set to ≤0.05, where *p* is the probability that the observed match is a random event. Proteins and their supporting peptides were obtained and analysed.

### Availability of supporting data

The raw reads of *T. pisana* mRNA are deposited in the NCBI GenBank as Sequence Read Archive (SRA) under the following accession numbers: SRP056280.

## Additional files

Additional file 1: Figure S1. Length distribution of unigenes. **Figure S2.** Graphs showing relative transcript abundance of top 50 transcripts in each *Theba pisana* transcriptome. **Figure S3.** Graph showing relative transcript abundance for *Theba pisana* neuropeptides identified. **Figure S4.** LC-MS/MS spectra showing peptides matching FFamide, NKY, sCAP and insulin precursors. **Figure S5.** *Theba pisana* insulin-like precursors annotated with features characteristic of insulin peptides. Yellow, signal peptide; blue, bioactive insulin; pink, cysteine residues; green, putative amidated glycine; red, cleavage sites. **Figure S6.** *Theba pisana* prohormone convertases, PC1 and PC2. Underline, signal peptide; red, catalytic region; boxed, conserved catalytic residues. Additional file 2: Table S1. Transcriptome annotations for *Theba pisana* CNS, muscle and hepatopancreas. Additional file 3: Table S2. Identification and annotation of secreted peptides within the CNS. All putative secreted peptides (Tab 1), gene ontology of secreted peptides (Tab 2), annotation of FPKM 10–100 (Tab 3) and FPKM >100 (Tab 4). Additional file 4:
**List of** ***Theba pisana*** **neuropeptides and prohormone convertases.**

## Abbreviations

CNS: Central nervous system
COG: Cluster of orthologous groups
CCAP: Crustacean cardioactive peptide
FPKM: Fragments per kilobase of exon region in a given gene per million mapped fragments
GDH: Glutamate dehydrogenase
GO: Gene ontology
MS: Mass spectrometry
NKY: Neuropeptide Y
PC: Prohormone convertase
SCP SCAP: Small cardioactive peptide
USC: University of the Sunshine Coast
