# Supplementary material for: Molecular insights into land snail neuropeptides through transcriptome and comparative gene analysis
Source: BMC Genomics. 2015 Apr 17;16(1):308. doi: 10.1186/s12864-015-1510-8 (PMC4408573; doi:10.1186/s12864-015-1510-8)
Supplement: Additional file 1: Figure S1. — Length distribution of unigenes. Figure S2. Graphs showing relative transcript abundance of top 50 transcripts in each Theba pisana transcriptome. Figure S3. Graph showing relative transcript abundance for Theba pisana neuropeptides identified. Figure S4. LC-MS/MS spectra showing peptides matching FFamide, NKY, sCAP and insulin precursors. Figure S5. Theba pisana insulin-like precursors annotated with features characteristic of insulin peptides. Yellow, signal peptide; blue, bioactive insulin; pink, cysteine residues; green, putative amidated glycine; red, cleavage sites. Figure S6. Theba pisana prohormone convertases, PC1 and PC2. Underline, signal peptide; red, catalytic region; boxed, conserved catalytic residues. [file 12864_2015_1510_MOESM1_ESM.docx]

**Additional files**

**Figure S1.** Length distribution of unigenes derived from a combined transcritpme assembly of *Theba pisana* CNS, foot muscle and hepatopancreas.

**Figure S2.** Graphs showing relative transcript abundance of top 50 transcripts in each *Theba pisana* transcriptome.

**Figure S3.** Graph showing relative transcript abundance for *Theba pisana* neuropeptides identified.

**Figure S4.** LC-MS/MS spectra showing peptides matching FFamide, NKY, sCAP and insulin precursors.

**Figure S5.** *Theba pisana* insulin-like precursors annotated with features characteristic of insulin peptides. Yellow, signal peptide; blue, bioactive insulin; pink, cysteine residues; green, putative amidated glycine; red, cleavage sites.

**Figure S6.** *Theba pisana* prohormone convertases, PC1 and PC2. Underline, signal peptide; red, catalytic region; boxed, conserved catalytic residues.


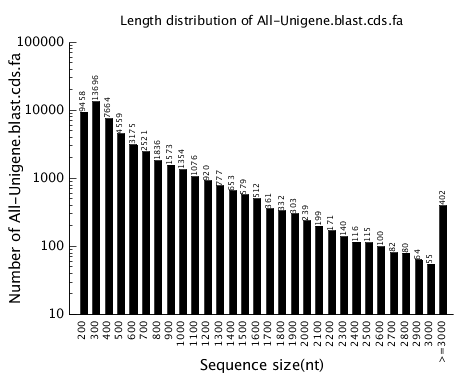


**Figure S1.** Length distribution of unigenes derived from a combined transcritpme assembly of *Theba pisana* CNS, foot muscle and hepatopancreas.


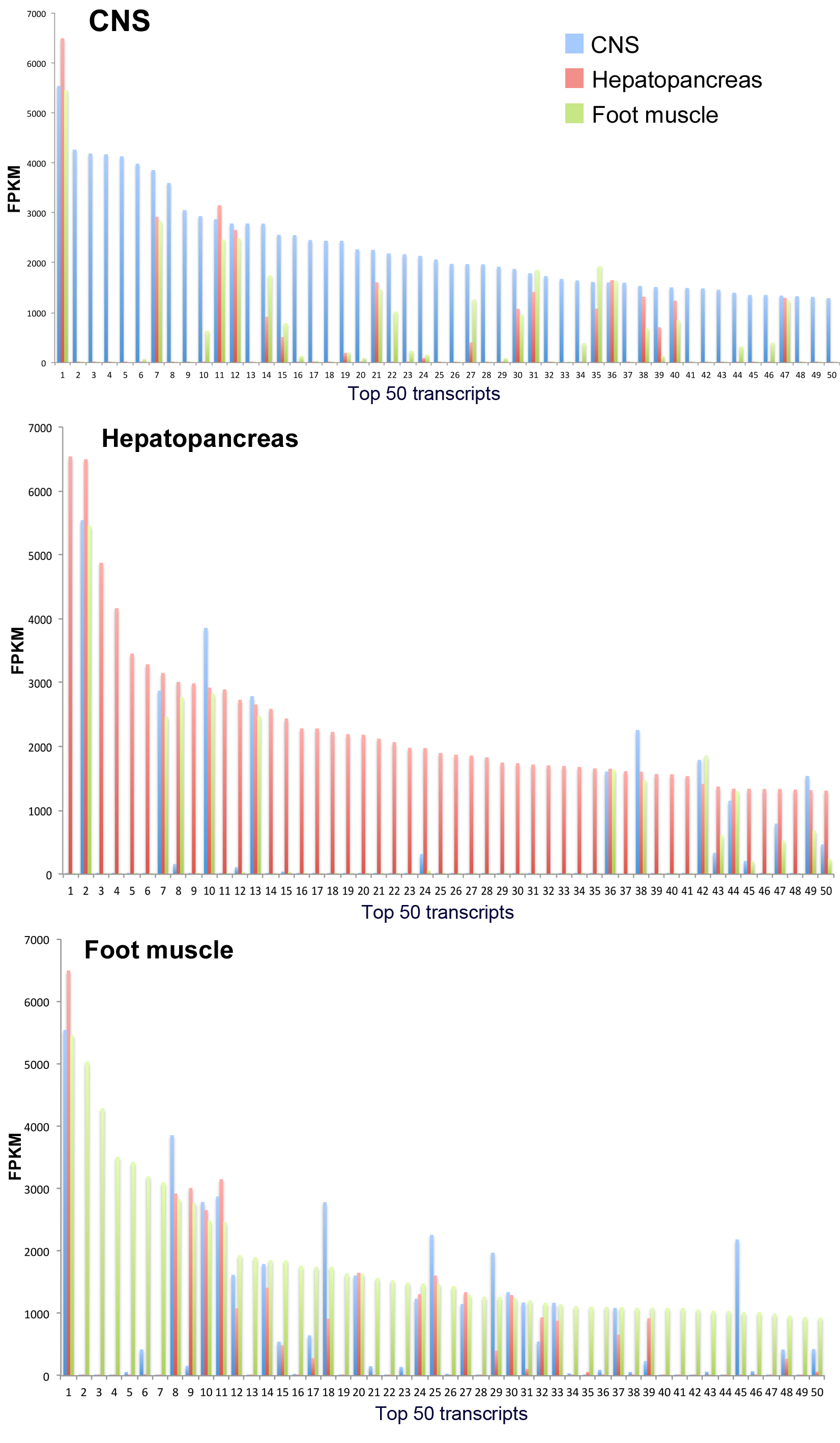


**Figure S2.** Graphs showing transcript abundance of top 50 transcripts in each *Theba pisana* transcriptome. Numbers for each correspond to transcripts listed in **Table S2**.


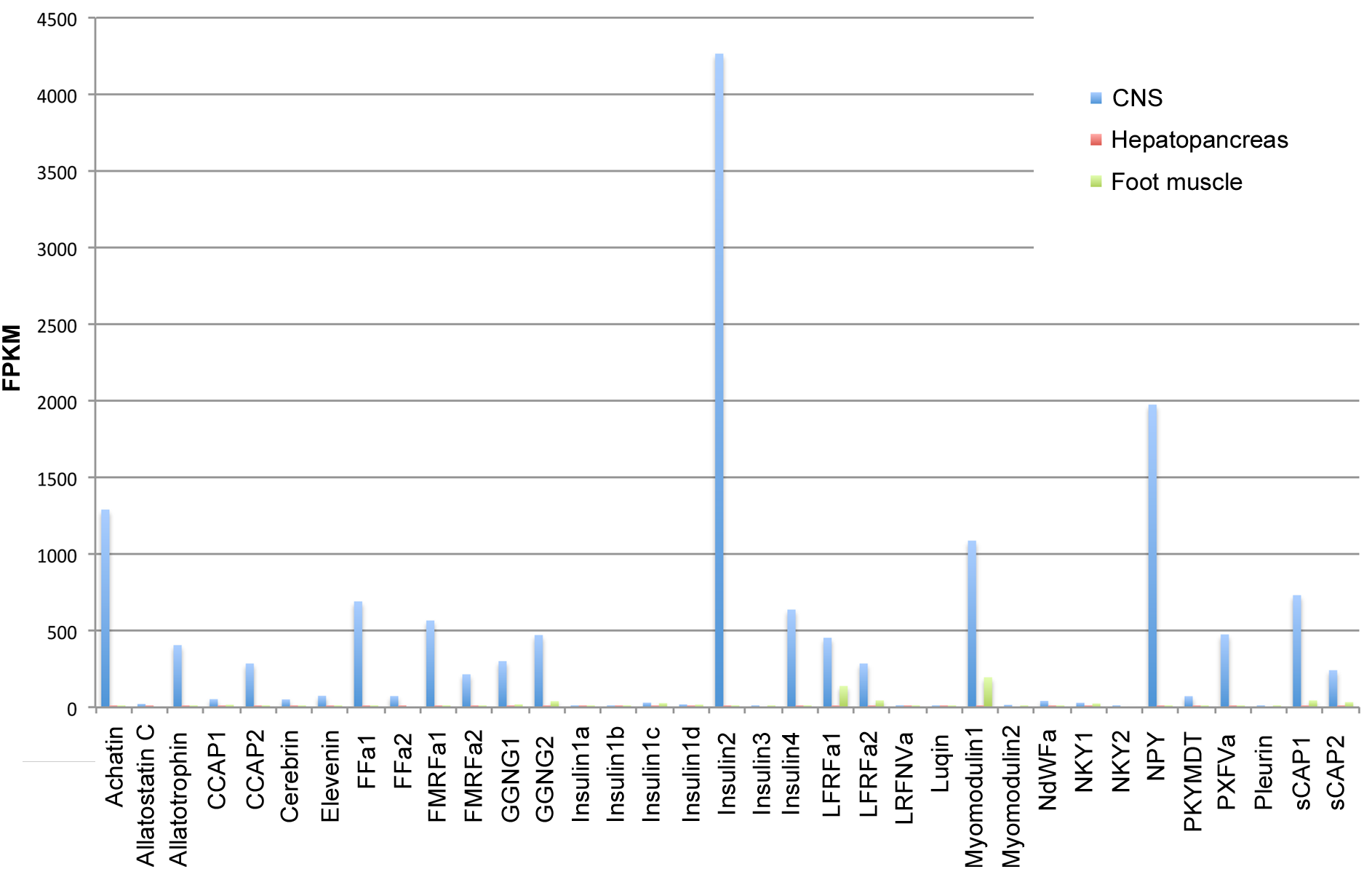


**Figure S3.** Graph showing transcript abundance for *Theba pisana* neuropeptides identified.

Tpi-Ffamide-1


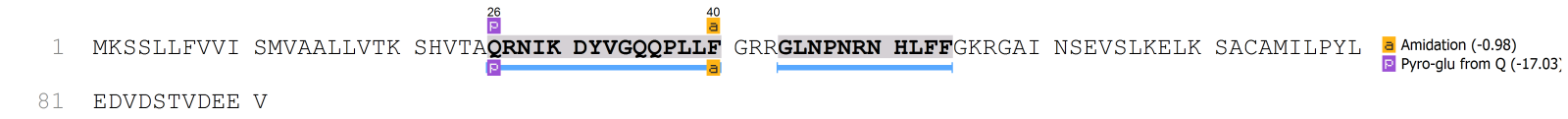


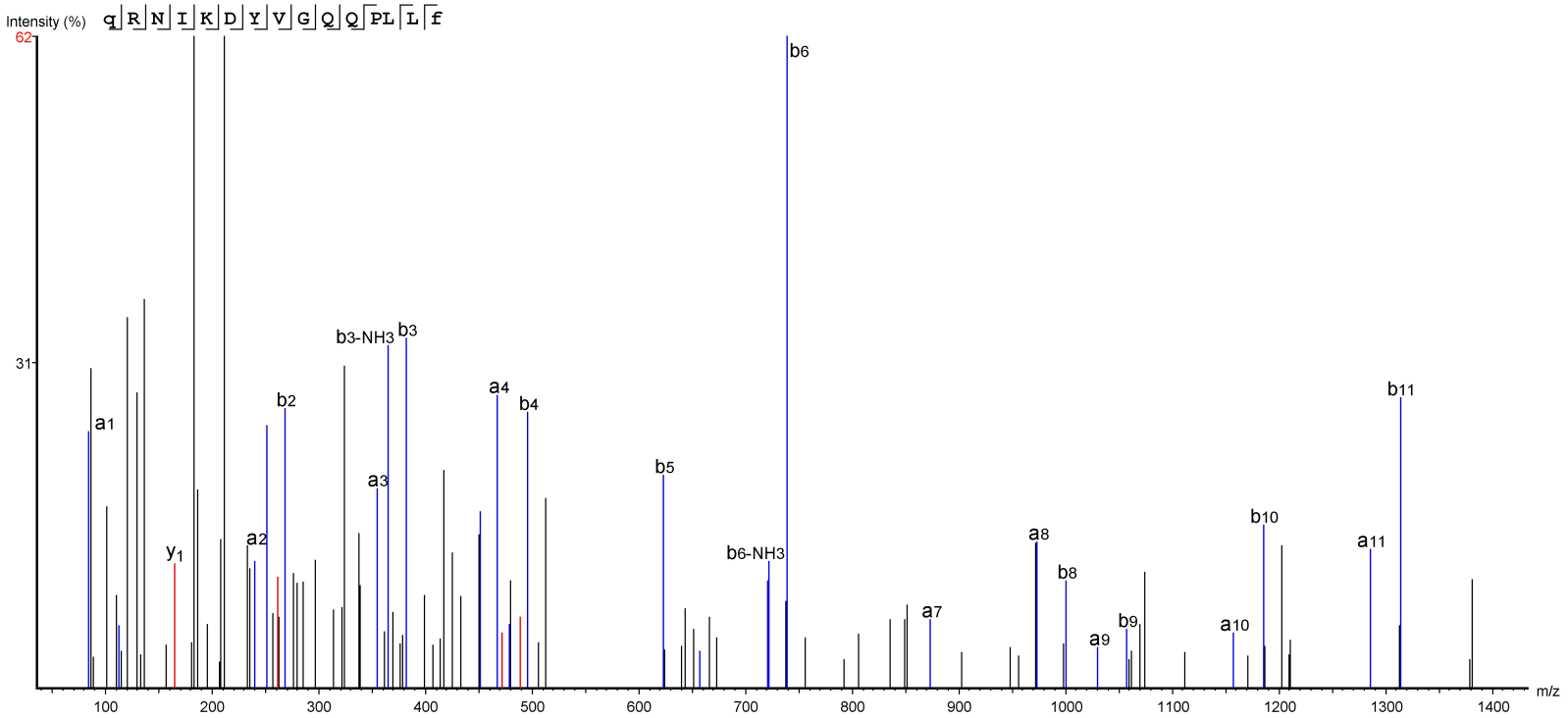


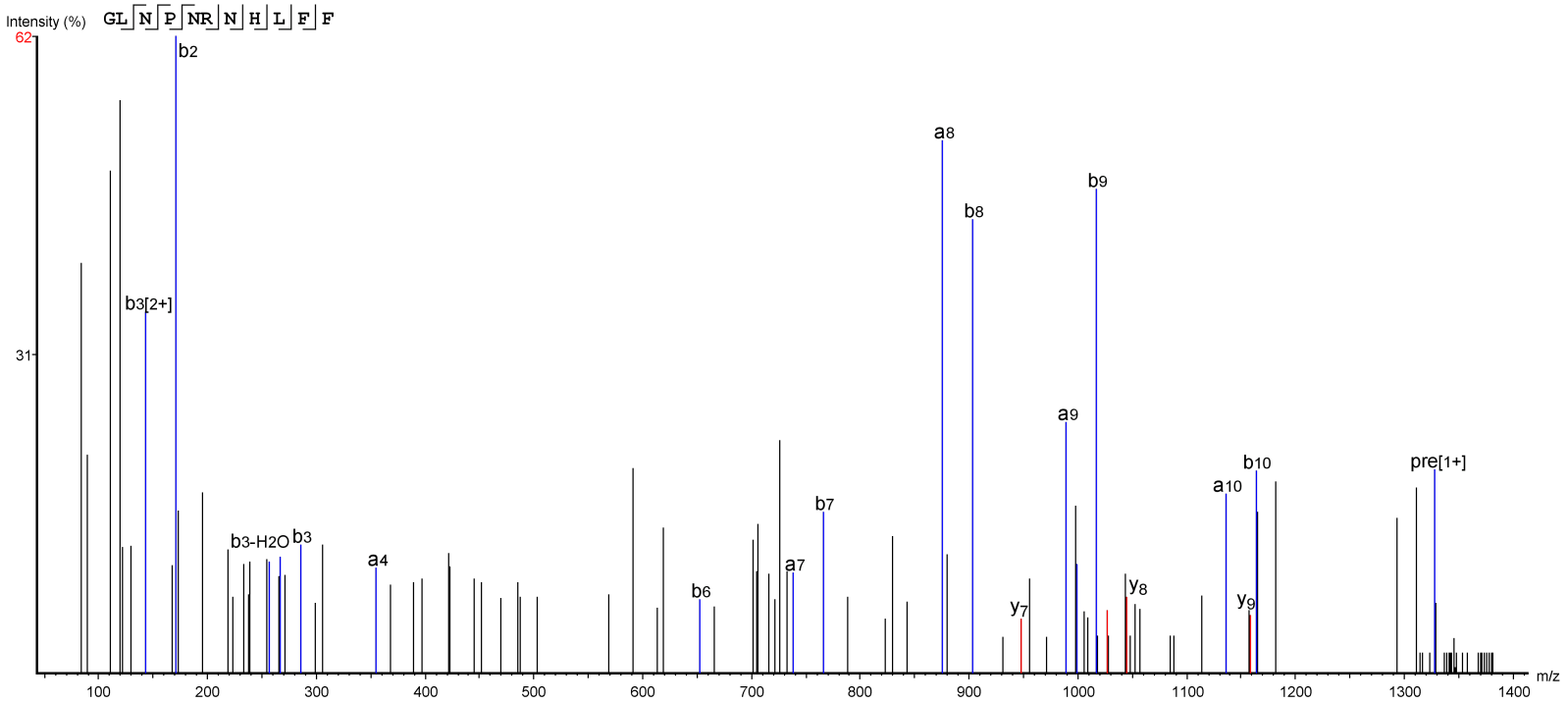


Tpi-NKY-1


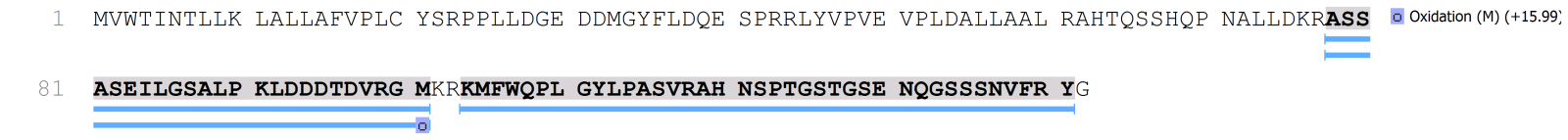


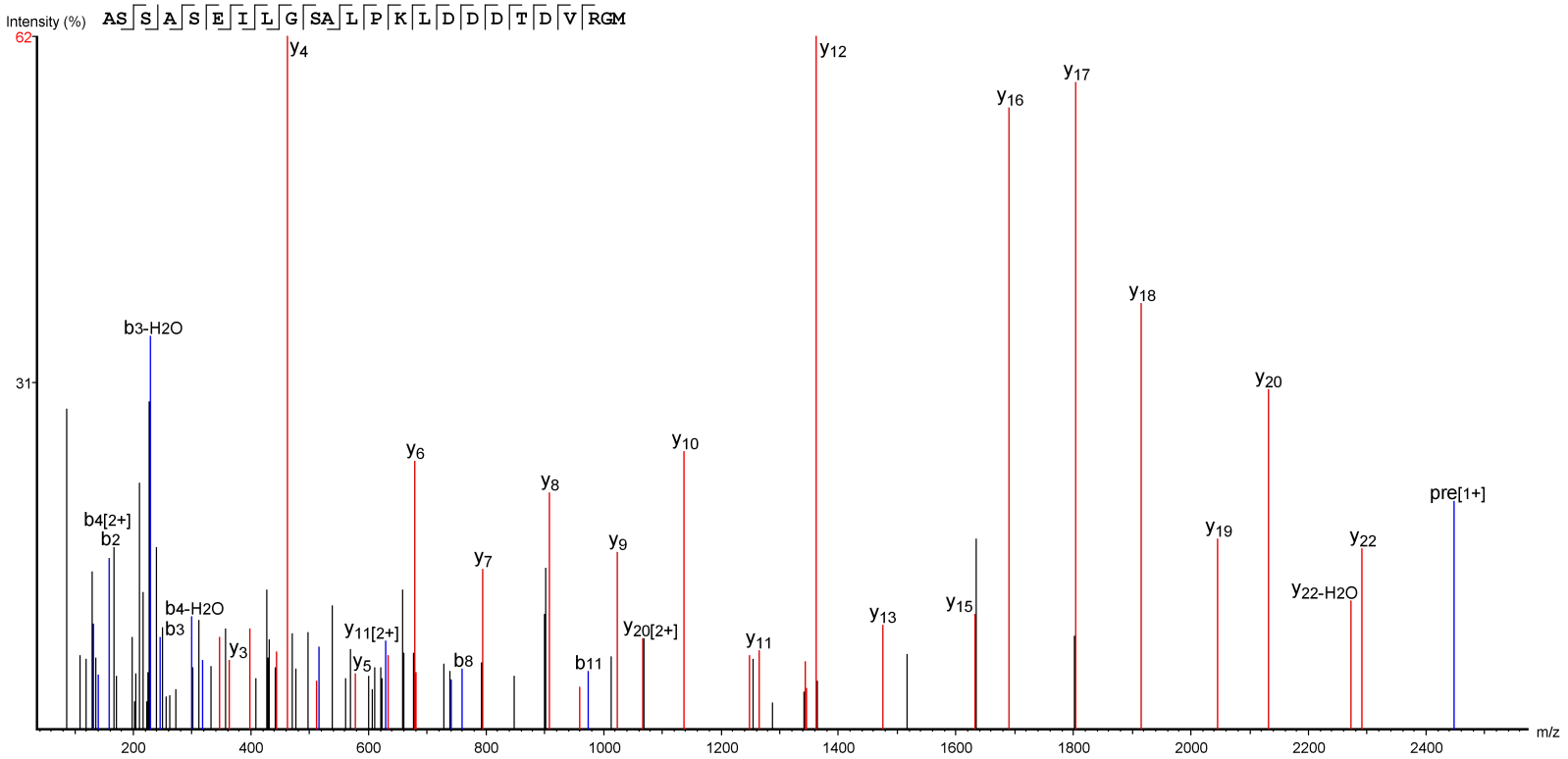


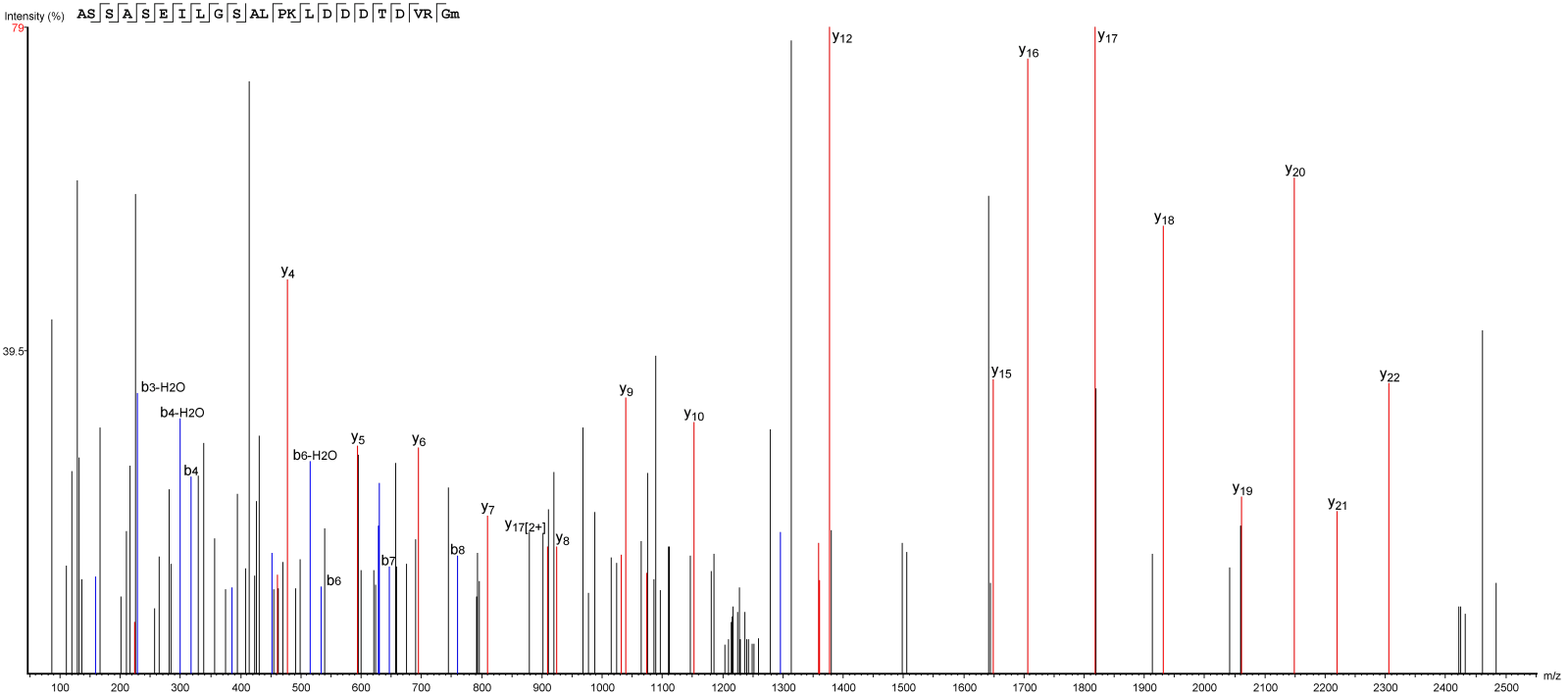


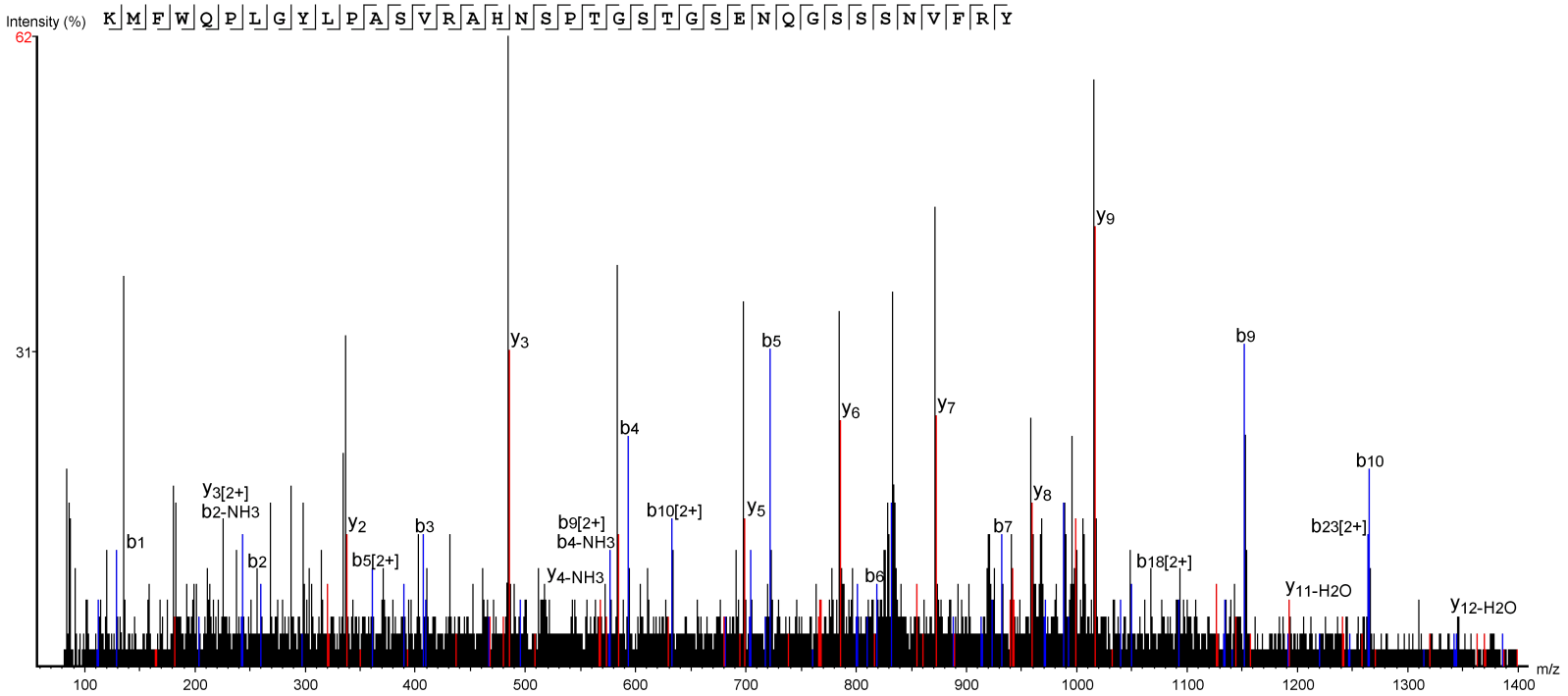


Tpi-sCAP-1


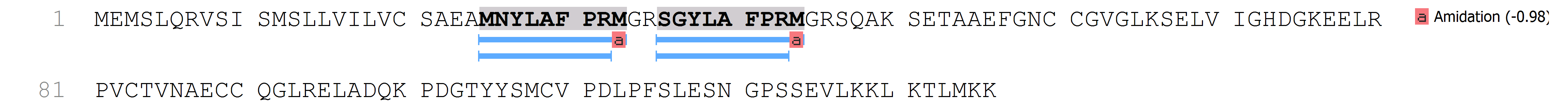


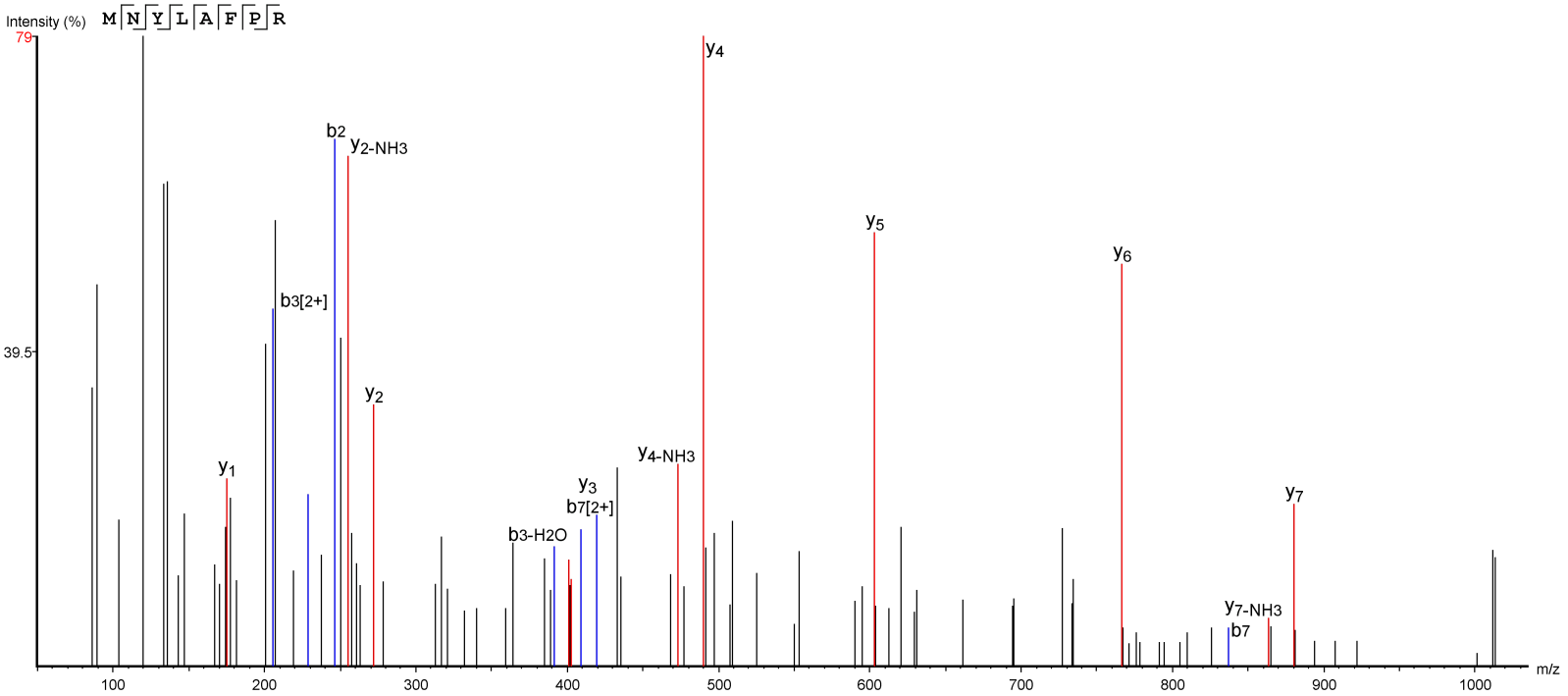


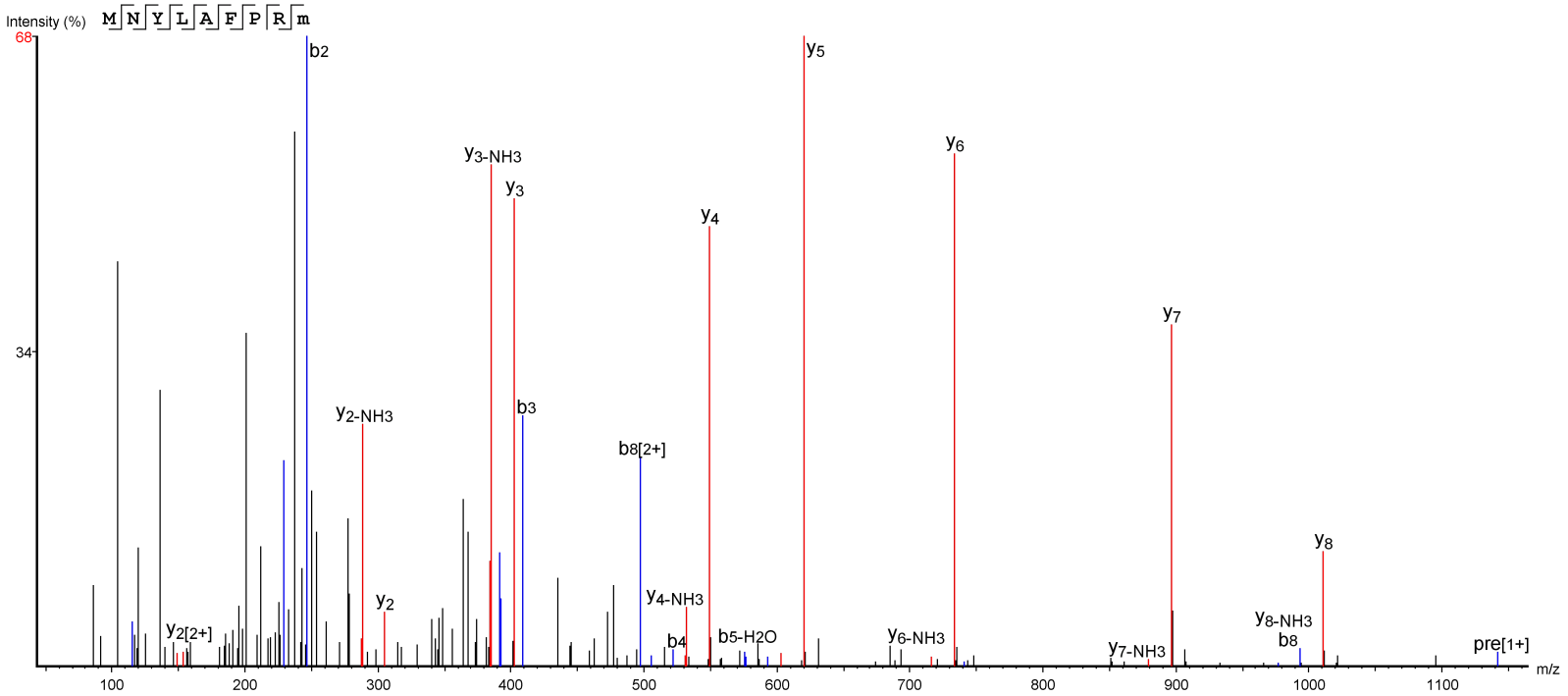


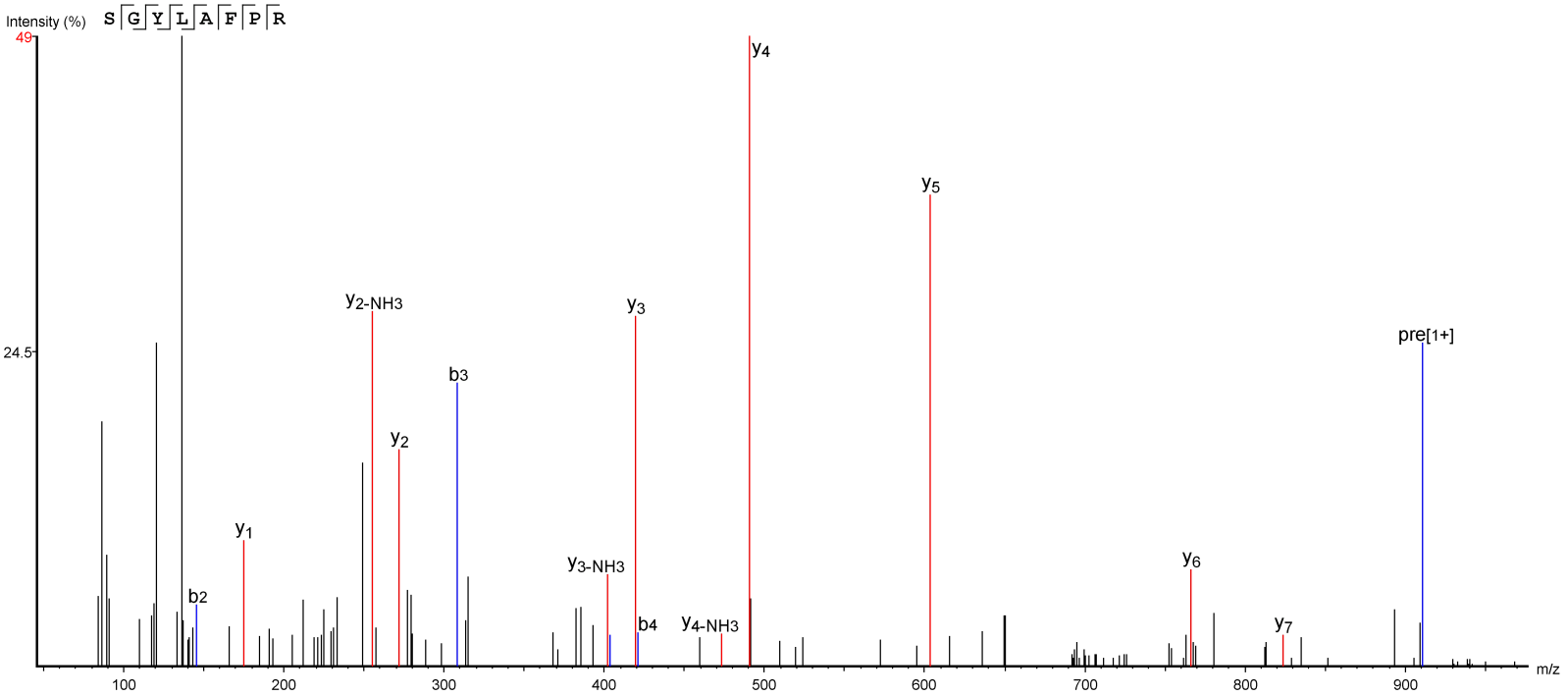


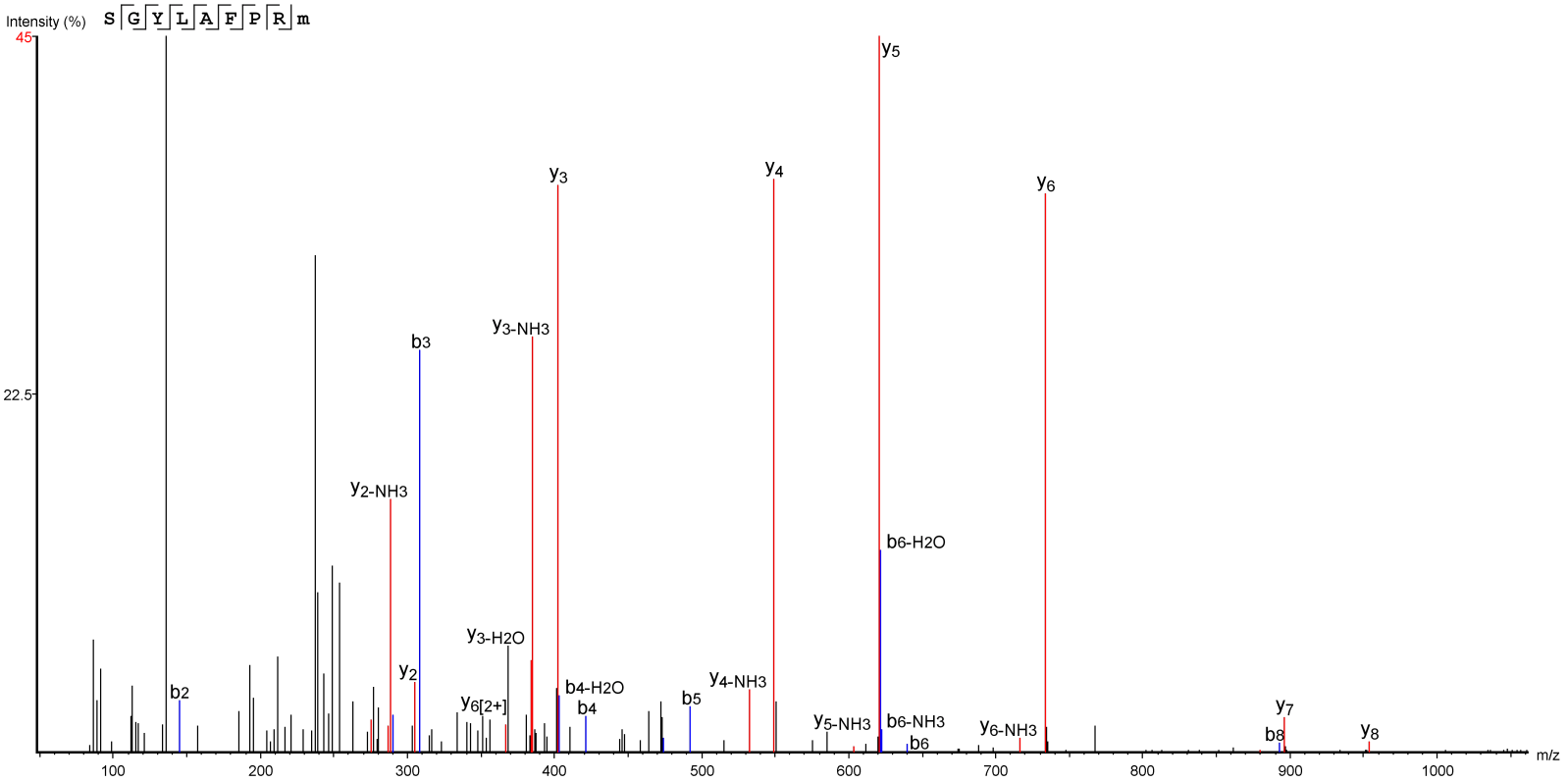


Tpi-insulin2


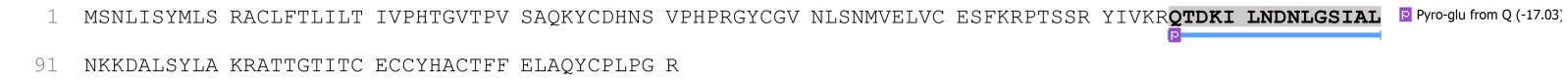


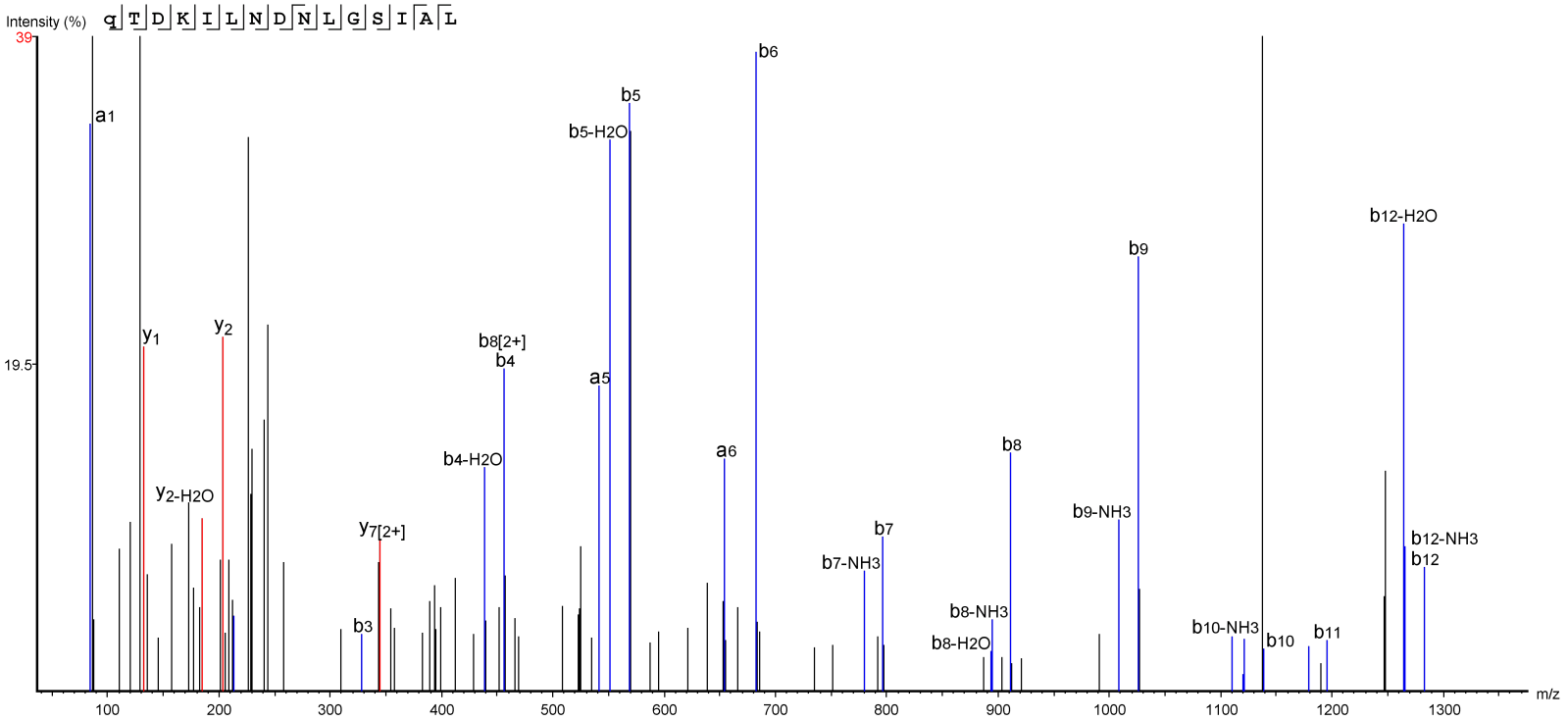


**Figure S4.** LC-MS/MS spectra showing peptides matching FFamide, NKY, sCAP and insulin precursors.

>Tpi-insulin1a

MVRNVEYYTTLTVALIAINLAIHQVQGQRRTCSLVARPHPNGYCGERLAQAHSNICFLLRRTYPHLFPMSKRSVPNESHISQSLDSLPDWELDGDESTRYPDSKSDFPPTLEDNSYLLSLLDTSDELADTSDEQADTSDEQADTSDEQADTSDEQADTSERPTASAFINFLEKRNSRRKRSLVCECCYAPCSFRIIARYC

>Tpi-insulin1b

MVRNVEYYTTLTVALIAINLAIHQVQGQRRTCSLVARPHPNGYCGERLAQAHSNICFLLRRTYPHLFPMSKRSVPNESHISQSLDSLPDWELDGDESTRYPDSKSDFPPTLEDNSYLLSLLDTSDELADTSDEQADTSDEQADTSDEQADTSERPTASAFINFLEKRNSRRKRSLVCECCYAPCSFRIIARYC

>Tpi-insulin1c

MVRNVEYYTTLTVALIAINLAIHQVQGQRRTCSLVARPHPNGYCGERLAQAHSNICFLLRRTYPHLFPMSKRSVPNESHISQSLDSLPDWELDGDESTRYPDSKSDFPPTLEDNSYLLSLLDTSDELADTSDEQADTSDEQADTSERPTASAFINFLEKRNSRRKRSLVCECCYAPCSFRIIARYC

>Tpi-insulin1d

MVRNVEYYTTLTVALIAINLAIHQVQGQRRTCSLVARPHPNGYCGERLAQAHSNICFLLRRTYPHLFPMSKRSVPNESHISQSLDSLPDWELDGDESTRYPDSKSDFPPTLEDNSYLLSLLDTSDELADTSDEQADTSERPTASAFINFLEKRNSRRKRSLVCECCYAPCSFRIIARYC

>Tpi-insulin2

MSNLISYMLSRACLFTLILTIVPHTGVTPVSAQKYCDHNSVPHPRGYCGVNLSNMVELVCESFKRPTSSRYIVKRQTDKILNDNLGSIALNKKDALSYLAKRATTGTITCECCYHACTFFELAQYCPLPGR

>Tpi-insulin3

MSGNADHCAALTCILLTFTLAVNQGQGQKRSCDLLSRPHPNGICGSMLAQVHENVCFLMRQAYPHFFPLRKRSPVLGDEDNHGGFLLPPLKNVLVSGDGYRVGPAVVGDTPFSLRRNSNAISPRKTFGLPGDIPQDQSENPIQRVLNKRNARSRSLVCECCYGPCTRRILASYC

>Tpi-insulin4

MASLVKVCLGVAAICVLVDVIVSQGMTDTENRFIADTTNRFSRLSATELLNAWHTECHRRCNYQLTWHVEIACRFDPYRIQGRRRRSIEKPRNLTNILRTTSDVKVTQNDVNASQPAYPKDPPQFMPRNSAMSFLKDHSYKRKKRDVSISRECCRNKACSWEEFAEYCQGHSRRASDRDSVCTYD

**Figure S5.** *Theba pisana* insulin-like precursors annotated with features characteristic of insulin peptides. Yellow, signal peptide; blue, bioactive insulin; pink, cysteine residues; green, putative amidated glycine; red, cleavage sites.


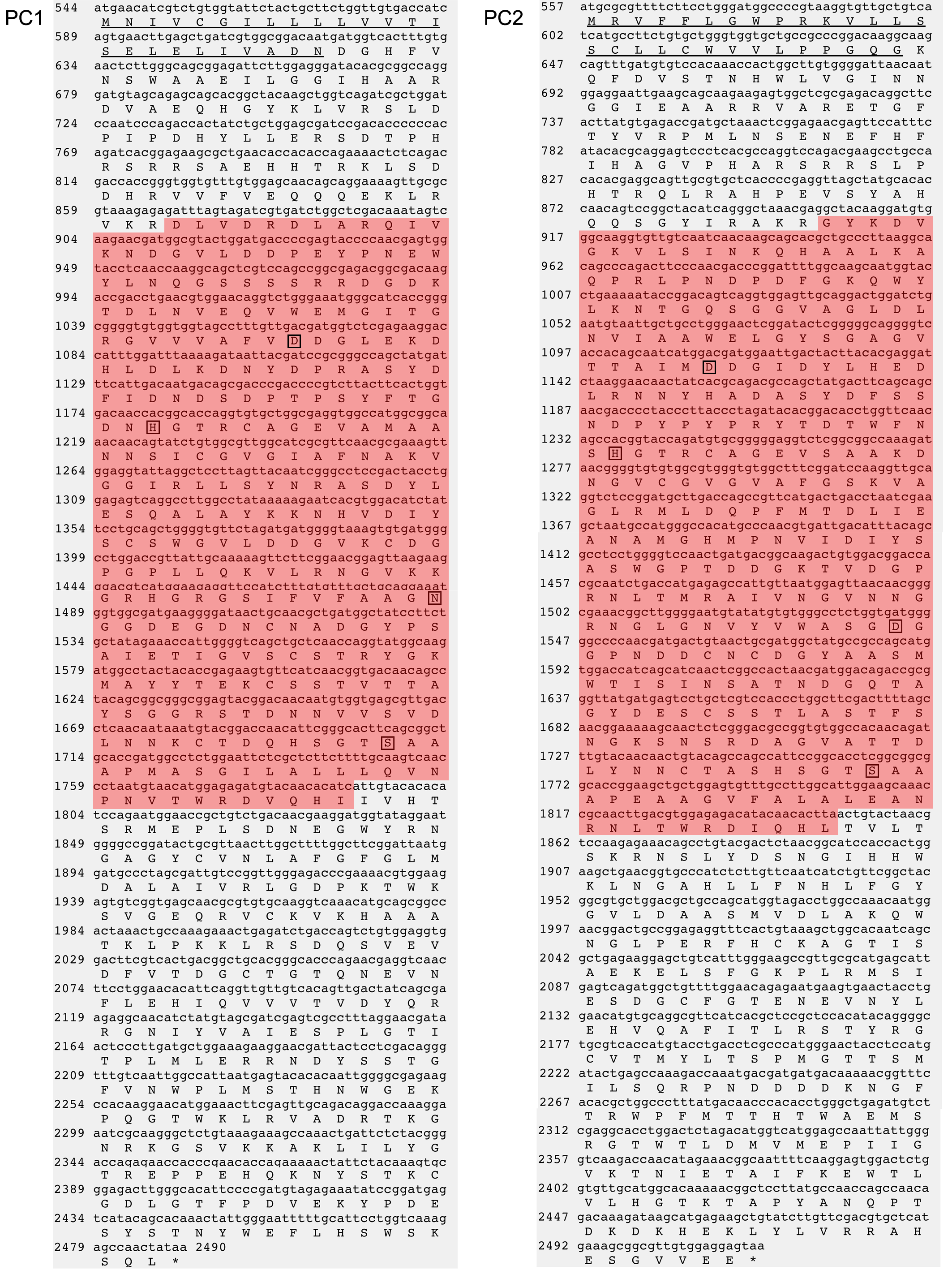


**Figure S6.** *Theba pisana* prohormone convertases, PC1 and PC2. Underline, signal peptide; red, catalytic region; boxed, conserved catalytic residues.
